# Comparative metabolomic and transcriptomic analysis revealing the role of salicylic acid for short-term response with arginine and myristic acid for long-term tolerance under salt stress in watermelon (*Citrullus lanatus* L.)

**DOI:** 10.3389/fpls.2026.1823719

**Published:** 2026-06-26

**Authors:** Haobin Pan, Xiaohui Li, Yong Wang, Xiaojing Ma, Haohao Ma, Hengbin Luo, Wenkai Shang, Ningning Gao, Liyun Kang, Xiaoxing Dong, Zhixin Guo, Han Dong, Fengzhi Piao, Sen Wang, Shen Li, Weixing Zhao, Tao Zhang

**Affiliations:** 1 College of Horticulture, Henan Agricultural University, Zhengzhou, China; 2 Horticultural Research Institute of Henan Academy of Agricultural Sciences, Zhengzhou, China; 3 Institute of Urban Agriculture, Chinese Academy of Agricultural Sciences, Chengdu, China; 4 Cash Crop Promotion Station of Henan Province, Zhengzhou, China

**Keywords:** *Citrullus lanatus* L., salt stress, salicylic acid, arginine, myristic acid, metabolome, transcriptome

## Abstract

**Introduction:**

Watermelon (*Citrullus lanatus* L.) is an important horticultural crop worldwide. Meanwhile, watermelon has a moderately tolerant susceptibility to salinity, making it an excellent model crop for studying salt stress-induced response and tolerance mechanism.

**Methods:**

Two watermelon homozygous inbred lines named ‘HY17’ (Salt-sensitive) and ‘HY73’ (Salt-tolerant) were used as materials. Seedlings at three-leaf age were treated with 150 mM NaCl for 2 days and 25 days to imitate short-term and long-term salt stress, respectively.

**Results:**

Comparative metabolomic and transcriptomic analysis combined with phenotypic stress indexes analysis revealed that higher biosynthesis of salicylic acid derived from the phenylalanine metabolism pathway in ‘HY73’ than ‘HY17’ is important for short-term response to salinity. Arginine and myristic acid accumulation, which is responsible for osmotic adjustment, reactive oxygen species (ROS) scavenging, and alleviating membrane lipid peroxidation, play roles in the tolerance to long-term salt stress in watermelon.

**Discussion:**

Differentially expressed genes (DEGs) of aspartate aminotransferase (AST: Cla97C04G073110 and Cla97C11G220050) and alanine transaminase (ALT: Cla97C09G163470 and Cla97C07G141140) are crucial for arginine biosynthesis, and the fatty acyl-ACP thioesterase B gene (FATB: Cla97C06G119690) is the key for myristic acid biosynthesis for improving salt tolerance. In addition, K+/Na+ transportation-related DEGs, including the high-affinity K+ transporter gene (HKT: Cla97C06G123400) and low-affinity K+ transporter gene (KT: Cla97C10G197240), and key members in salt overly sensitive (SOS) pathway (SOS2/CIPK: Cla97C02G045030 and SOS3/CBL: Cla97C06G127530) were identified as responsible for regulating intracellular ionic homeostasis as salinity resistance. Hence, these results highlight the holistic resistant mechanisms of short-term response and long-term tolerance under salt stress in watermelon.

## 1 Introduction

Watermelon (*Citrullus lanatus* L.) is an important horticultural crop with a moderately tolerant susceptibility to salinity (Romic et al., 2008). Salinity is one of the most severe environmental factors limiting the growth and productivity of crops and sustainable development in agriculture, especially in arid and semi-arid regions (Zörb et al., 2019). Improper irrigation practices and the excessive use of chemical fertilizers further aggravate soil salinity in modern agriculture; approximately 20%–30% of agricultural irrigated land is affected by salt accumulation (Liang et al., 2024; Sahab et al., 2021). Therefore, it is necessary to improve salt resilience to meet future watermelon production.

Excessive salt accumulation in soil solution reduces water and nutrient uptake, which leads to osmotic stress, ion toxicity, photosynthesis inhibition, oxidative damage, and nutrient imbalance (Ma et al., 2026; Zhou et al., 2024). At the physiological level, excess soluble salts (such as Na^+^) reduce the water potential around the root in the soil, causing osmotic stress, thereby limiting water uptake of plant (van Zelm et al., 2020). Concurrently, salinity induced osmotic stress, triggering stomatal closure and limiting the available carbon dioxide (CO_2_) and fixation for plant photosynthesis cycle (Lawson and Vialet-Chabrand, 2019; Niu et al., 2018). Elevated Na^+^ uptake disrupts cellular Na^+^/K^+^ homeostasis by competing with K^+^, since Na^+^ and K^+^ share the transport system. This ionic imbalance will disturb metabolic processes for plant growth (Niu et al., 1995; Rodríguez-Navarro and Rubio, 2006). In addition, Cl^-^ accumulation may induce plasma membrane lipid peroxidation, inhibit NO_3_^−^ uptake, and destroy mitochondrial ultrastructure (Ashraf et al., 2018; Glass and Siddiqi, 1985; Rodríguez-Navarro and Rubio, 2006). On the other hand, prolonged exposure to salt stress triggers excessive reactive oxygen species (ROS) production through electron leakage in chloroplasts and mitochondria, resulting in oxidative damage to cellular lipids, proteins, and nucleic acids (Gill and Tuteja, 2010; Miller et al., 2010). In summary, salt stress exerts multilevel stacking effects on plant survival.

Watermelon has a relative tolerance to salt stress, so it serves as an excellent model crop for studying salt stress-induced responses (Liu et al., 2023). Previous studies have reported that watermelon seedlings will appear to have inhibited growth at a salt level of NaCl higher than 75 mM, and the growth and development of watermelon seedlings will be significantly suppressed when the NaCl concentration is higher than 150 mM, which can seriously restrict the yield and quality of watermelon production (Chen et al., 2024). A study on watermelon seedlings treated with 150 mM NaCl revealed that salt stress significantly decreased the biomass accumulation and K^+^ level in roots and leaves, while significantly increasing Na^+^ and Cl^-^ uptake, and increasing the malondialdehyde (MDA) content. Notably, the salt-tolerant watermelon variety exhibited higher K^+^ absorption, lower Cl^-^ level, and lower MDA content in roots and leaves compared to the salt-sensitive watermelon variety under 150 mM NaCl salt stress (Zhu et al., 2022). However, the metabolomic and transcriptomic mechanisms of short-term response and long-term tolerance under salt stress in watermelons have not been revealed. Therefore, seedlings of two watermelon cultivars ‘HY17’ (salt-sensitive) and ‘HY73’ (salt-tolerant) were used as materials, and a concentration of 150 mM NaCl was used as treatment to elucidate the differential mechanisms between short-term and long-term salt stress through comparative transcriptome and metabolome, aiming to investigate and provide new insights for watermelon salt-tolerant breeding.

## 2 Materials and methods

### 2.1 Plant materials and treatments

Watermelon homozygous inbred lines named ‘HY17’ (salt-sensitive) and ‘HY73’ (salt-tolerant) provided by the Horticulture Research Institute of Henan Academy of Agricultural Sciences (Zhengzhou, China) were used as materials. Watermelon seedings were cultivated with vermiculite using 50% Hoagland’s nutrient solution under 12 h light (200 µmol m^-^² s^-^¹) and 12 h dark at a temperature of 25 °C/20°C and 60% relative humidity. Seedlings were treated with a 150 mM NaCl solution from the three-leaf age for 2 and 25 days to imitate short-term and long-term salt stress, respectively.

### 2.2 Phenotypic analysis under salt stress

Growth parameters, including plant height and fresh and dry weights of shoots and roots of watermelon seedlings, were measured after 25 days of salt treatment. Plant height was measured using a ruler. The fresh and dry weights of shoots and roots were determined as previously described by Zhang et al. (2022). Root shape parameters were measured by a plant root analysis system (REGENT Instruments WinRHIZO, Canada). Root vitality was assessed by the triphenyltetrazolium chloride (TTC) method (Clemensson-Lindell, 1994). There were three independent biological replicates per treatment for each indicator. The salt stress index of each parameter was calculated as follows:

Stress index = (Value of control – Value of NaCl)/Value of control

Chlorophyll fluorescence of maximal photochemical efficiency of PS-II (*F*v/*F*m) was measured using a chlorophyll fluorescence imager (Photon Systems Instruments FluorCam, Czech Republic) after plants were kept under dark conditions for 30 min. In addition, the chlorophyll content in the leaves was detected by the ethanol-extracted spectrophotometric method (Ritchie, 2006). Three independent biological replicates were set for each treatment.

Hydrogen peroxide (H_2_O_2_) and superoxide (O_2_^•−^) accumulation in leaves was indicated using 3,3′-diaminobenzidine (DAB) and nitroblue tetazolium (NBT) histochemical staining, respectively, according to our previous study (Ma et al., 2025b). Relative electrical conductivity determination of leaves was performed as previously described (Zhang et al., 2022). MDA content in leaves and roots was determined by the thiobarbituric acid (TBA) colorimetric method using a commercial kit (Michy Biomedical Technology M0106A, China). Three independent biological replicates were set for each treatment.

Antioxidant enzyme activities in leaves and roots were determined using commercial kits for catalase (CAT) (Michy Biomedical Technology M0104A, China), peroxidase (POD) (Michy Biomedical Technology M0105A, China), and superoxide dismutase (SOD) (Michy Biomedical Technology M0102A, China), respectively. Three independent biological replicates were set for each treatment.

### 2.3 Na^+^ and K^+^ content determination

Determination of Na^+^ and K^+^ content was according to the method described by Zhang et al. (2022). Briefly, powder of 100 mg dried shoot or root samples of watermelon seedlings was digested with nitric acid, then the Na+ and K+ content was determined using an inductively coupled plasma-optical emission spectrometer (ICP-OES) (Thermo Fisher Scientific iCAP 7200 HS Duo, USA).

### 2.4 Metabolome analysis

Metabolome detection was performed by Personalbio Technology Co., Ltd. (Shanghai, China). Lyophilized root tissue (20 mg) was vortexed using 1 mL of extraction solution [MeOH: ACN:H_2_O = 2:2:1 (v/v)] for 30 s. Using deuterium as the relative quantitative internal marker. Then, the mixed samples were homogenized (35 Hz, 4 min) and sonicated at 4°C for 5 min twice. Next, the samples were incubated at −40°C for 1 h to precipitate proteins. Then, the samples were centrifuged at 12,000 rpm at 4°C for 15 min. The supernatant was collected for analysis using a UHPLC system (Thermo Fisher Scientific Vanquish, USA) with a Phenomenex Kinetex C18 chromatographic column (2.1 mm × 50 mm, 2.6 μm) coupled with an Orbitrap Exploris 120 mass spectrometer (Thermo Fisher Scientific Orbitrap MS, USA). Mobile phase A: 0.01% acetic acid water solution; mobile phase B: IPA: CAN = 1:1 (v/v). The auto-sampler temperature was set to 4°C, and the injection volume was 2 μL.

The differentially accumulated metabolites (DAMs) were screened with the thresholds of fold change > 1, *p*-value < 0.05, and VIP > 1. Cluster analysis of DAMs between sample groups is exhibited in **Supplementary Figure 6**. Kyoto Encyclopedia of Genes and Genomes (KEGG) enrichment analysis for DAMs was tested using the Benjamini–Hochberg method.

### 2.5 Transcriptome sequencing and analysis

Root tissues of ‘HY17’ and ‘HY73’ watermelon seedlings treated with 150 mM NaCl solution for 2 and 25 days were used for RNA extraction and transcriptome sequencing with an Illumina platform (NovaSeq 6000 System, USA) at Personalbio Technology Co., Ltd. (Shanghai, China). The mapped efficiency of the RNA-seq data against the watermelon genome 97103_genome_v2.5 (http://cucurbitgenomics.org/v2/ftp/genome/watermelon/97103/v2.5) using HISAT2 ranged from 97.81% to 98.11%. The raw sequencing data are available on the NCBI SRA (https://www.ncbi.nlm.nih.gov/sra) with the BioProject accession number of PRJNA1429590.

The differentially expressed genes (DEGs) were analyzed using the DESeq (v1.38.3) package with the screening thresholds of |log_2_fold change| > 1 and *p*-value < 0.05. Cluster analysis of DEGs between sample groups is exhibited in **Supplementary Figure 7**. KEGG and Gene Ontology (GO) enrichment analyses were performed using the topGO (v2.50.0) and ClusterProfiler (v4.6.0) packages, respectively. The standard of *p*-value < 0.05 was adopted for GO terms and KEGG pathways enrichment.

### 2.6 Statistical analysis

Significant differences among treatments were analyzed using one-way analysis of variance (ANOVA) by SPSS Statistics 22.0 software (IBM SPSS Inc., USA). Metabolome and transcriptome data analysis and visualization of KEGG and GO enrichment were performed on the online platform Personalbio GenesCloud (https://www.genescloud.cn). Bar charts were plotted by OriginPro 2021 software (OriginLab Corporation, USA). Heatmaps of gene expression were visualized by TBtools-II software after the TPM values were log_2_-transformed (Chen et al., 2023). K^+^/Na^+^ transportation and SOS pathway gene families were identified genome-wide using the Advanced HMMer Search plugin in TBtools-II software against the Pfam conserved domain for ClHKTs (PF02386), ClKTs (PF02705), SOS1 (ClNHXs) (PF00999), SOS2 (ClCIPKs) (PF00069 and PF03822), and SOS3 (ClCBLs) (PF13202 and PF13833).

## 3 Results

### 3.1 Physiological performance of ‘HY17’ and ‘HY73’ under short-term and long-term salt stress

**Figure 1:**
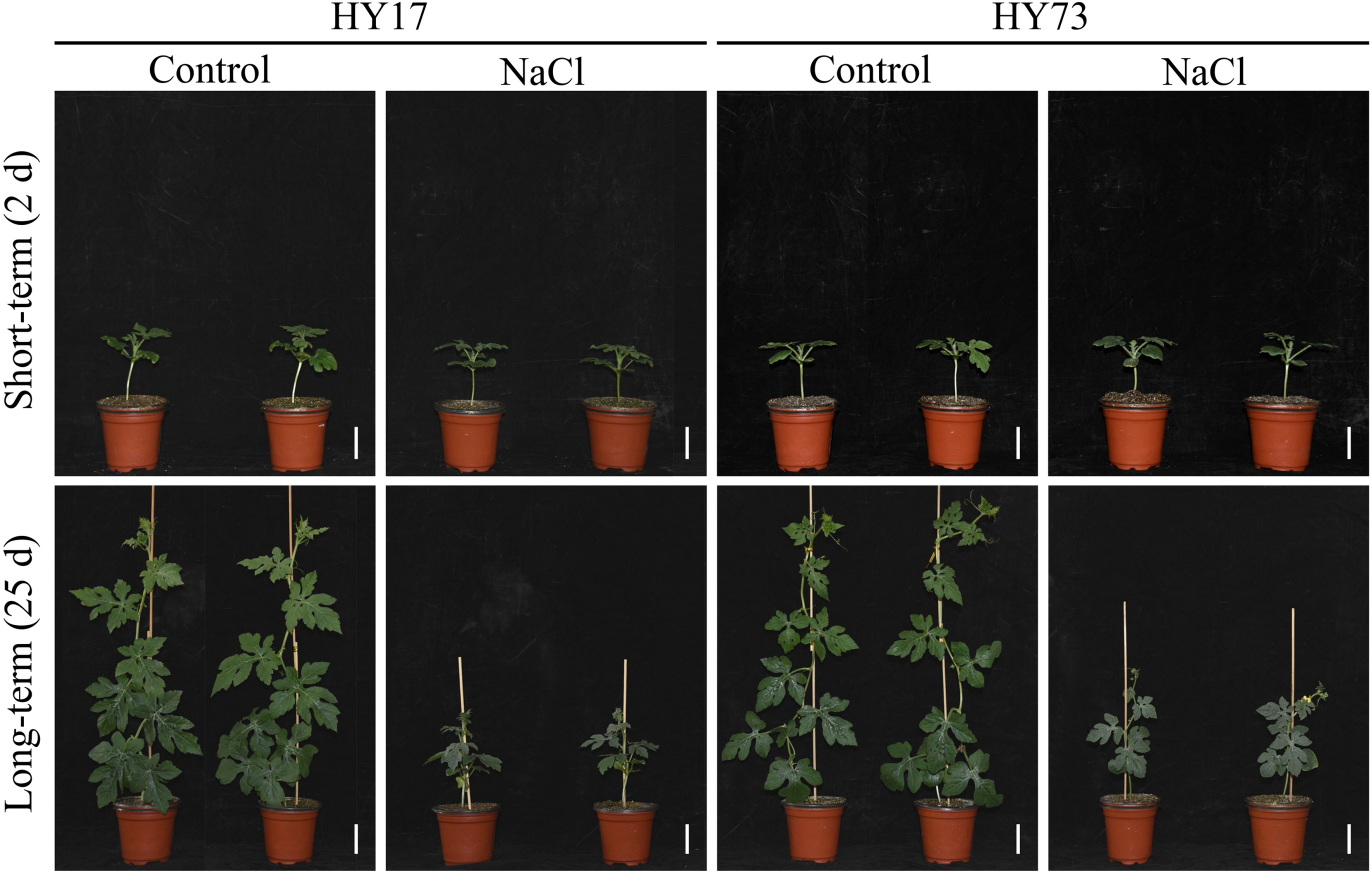
Performance of ‘HY17’ and ‘HY73’ watermelon seedlings under salt stress for 2 and 25 days. Bar = 5 cm.

Watermelon seedlings of ‘HY17’ and ‘HY73’ were treated with 150 mM NaCl for 2 and 25 days to imitate short-term and long-term salt stress, respectively. The phenotype of ‘HY17’ and ‘HY73’ between control and NaCl groups showed that ‘HY73’ has a higher plant growth rate than ‘HY17’ under 25 days of salt stress (**Figure 1**). The stress indexes of various plant growth parameters including plant height, shoot fresh weight, shoot dry weight, root fresh weight, and root dry weight of ‘HY73’ are all significantly higher than those of ‘HY17’ (**Table 1**). Moreover, ‘HY73’ exhibits more vigorous root growth than ‘HY17’; the total root length, total root volume, total root surface area, root average diameter, number of root tips, and root vitality of ‘HY73’ are all significantly higher than those of ‘HY17’ under long-term salt stress, indicating that the root growth greatly determines the salinity tolerance (**Table 1**; **Supplementary Figure 1**).

**Table 1:** The effect on plant growth of ‘HY17’ and ‘HY73’ watermelon seedlings under salt stress for 25 days.

Parameters	HY17			HY73		
	Control	NaCl	Stress index	Control	NaCl	Stress index
Plant height (cm)	37.68 ± 0.82b	8.98 ± 0.86d	0.76	41.15 ± 0.94a	14.58 ± 0.31c	0.65
Shoot fresh weight (g)	13.55 ± 1.17a	2.75 ± 0.24c	0.80	9.04 ± 0.34b	3.10 ± 0.15c	0.66
Shoot dry weight (g)	1.03 ± 0.60a	0.32 ± 0.03c	0.69	0.73 ± 0.05b	0.37 ± 0.03c	0.49
Root fresh weight (g)	1.40 ± 0.09a	0.43 ± 0.04d	0.69	1.16 ± 0.02b	0.58 ± 0.02c	0.50
Root dry weight (g)	0.060 ± 0.002a	0.027 ± 0.001b	0.55	0.054 ± 0.007a	0.030 ± 0.007b	0.44
Total root length (cm)	396.21 ± 12.67b	282.36 ± 6.54c	0.29	432.12 ± 11.65a	379.12 ± 11.63b	0.12
Total root volume (cm^3^)	4.16 ± 0.36b	1.04 ± 0.06d	0.75	5.71 ± 0.92a	2.33 ± 0.48c	0.59
Total root surface area (cm^2^)	139.09 ± 9.20b	60.01 ± 2.66d	0.57	165.62 ± 5.74a	120.16 ± 2.14c	0.27
Root average diameter (mm)	0.94 ± 0.02b	0.62 ± 0.05c	0.34	1.29 ± 0.12a	1.07 ± 0.11b	0.17
Number of root tips	3,027.33 ± 69.92a	1,663.07 ± 5.03c	0.45	3,002.33 ± 86.73a	2,185.73 ± 42.76b	0.27
Root vitality (μg·g^−1^·h^−1^)	92.37 ± 3.39a	41.27 ± 2.79c	0.55	77.25 ± 2.79b	45.74 ± 1.78c	0.41

Values are the means ± SD of three biological replicates (*n* = 3), and different letters represent significant differences at the level of *p* < 0.05. Stress index = (Value of control – Value of NaCl)/Value of control.

**Figure 2:**
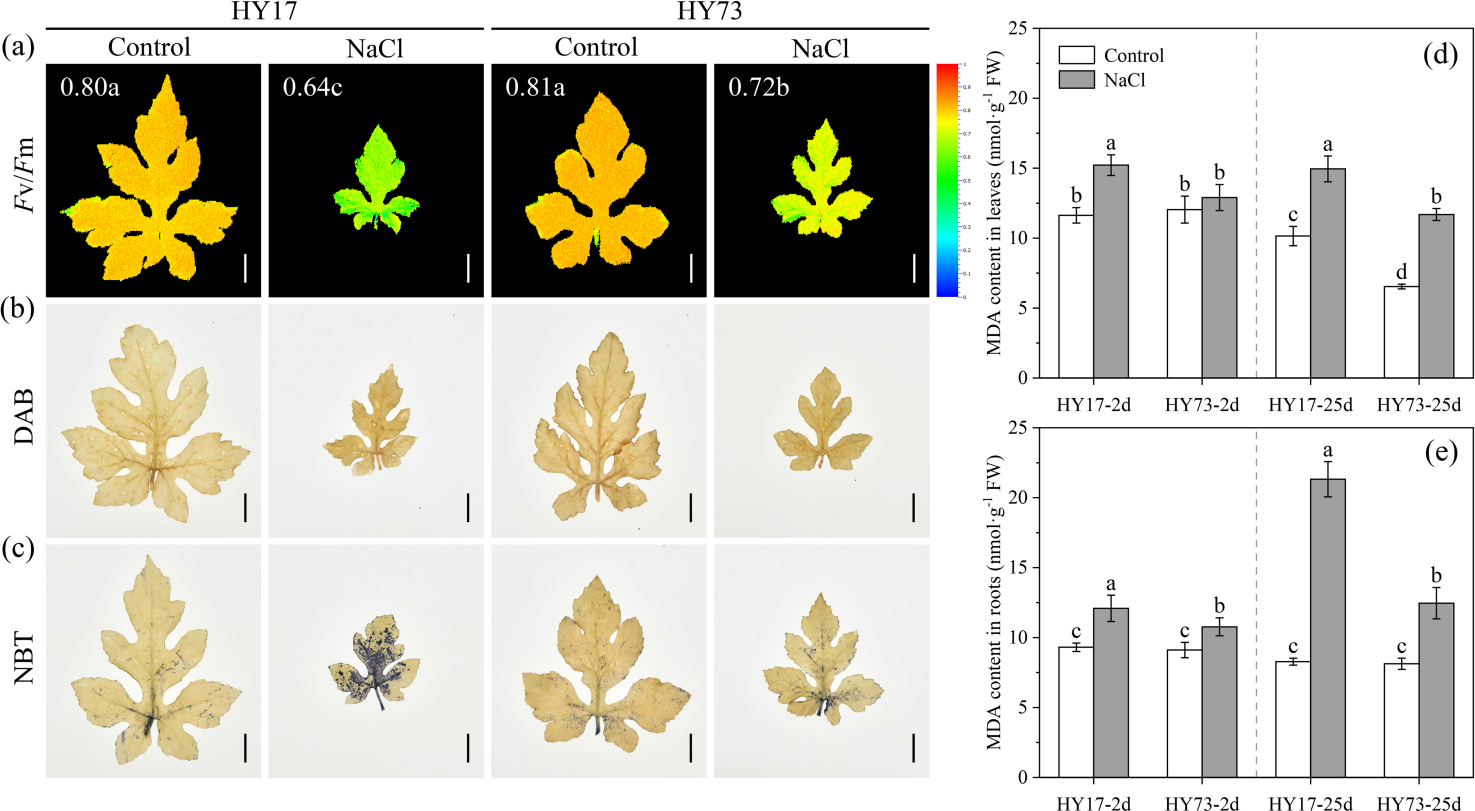
Photosynthetic chlorophyll fluorescence, oxidative damage, and membrane lipid peroxidation of ‘HY17’ and ‘HY73’ watermelon seedlings under salt stress. **(a)** Chlorophyll fluorescence of maximal photochemical efficiency of PS-II (*F*v/*F*m) of leaves. Bar = 2 cm. **(b)** 3,3′-Diaminobenzidine (DAB) staining for H_2_O_2_ accumulation in leaves. Bar = 2 cm. **(c)** Nitroblue tetrazolium (NBT) staining for O_2_^•−^ accumulation in leaves. Bar = 2 cm. **(d)** Malondialdehyde (MDA) content in leaves under salt stress for 2 and 25 days. **(e)** MDA content in roots under salt stress for 2 and 25 days. Values are the means ± SD of three biological replicates (*n* = 3), and different letters on each column indicate significant differences between means among different groups at the *p* < 0.05 level compared by one-way ANOVA with Duncan test.

In addition, oxidative damage extent and photosynthetic chlorophyll fluorescence indexes showed that ‘HY73’ accumulates less H_2_O_2_ and O_2_^•–^ (indicated by DAB and NBT staining, respectively) and a lower degree of membrane lipid peroxidation (indicated by MDA content) than ‘HY17’ in leaves under salt stress (**Figures 2b–d**). ‘HY73’ leaves showed significantly higher chlorophyll fluorescence (*F*v/*F*m) and chlorophyll content than ‘HY17’ leaves (**Figure 2a**; **Supplementary Figure 2**). The MDA content and relative electrical conductivity in roots indicated that ‘HY17’ experienced more severe membrane lipid peroxidation damage than ‘HY73’ in roots under long-term salt stress (**Figure 2e**; **Supplementary Figure 3**). However, the activities of ROS scavenging enzymes in ‘HY17’ and ‘HY73’ did not show consistent results. POD and SOD activities were induced, while CAT activities were suppressed in both cultivars under long-term salt stress in leaves (**Supplementary Figures 4a, c, e**). POD plays an important role for ROS resistance response to salinity in roots of ‘HY17’ and ‘HY73’ seedlings, but no significant differences were observed in CAT and SOD activities between the two cultivars in roots (**Supplementary Figures 4B, D, F**).

### 3.2 KEGG enrichment of comparative metabolomic analysis of ‘HY17’ and ‘HY73’ under short-term and long-term salt stress

A total of 183 DAMs (81 up-accumulated and 102 down-accumulated) were identified between the pairwise comparisons of HY17-NaCl-2d_vs_HY73-NaCl-2d, while 64 DAMs (23 up-accumulated and 41 down-accumulated) were identified between the pairwise comparisons of HY17-NaCl-25d_vs_HY73-NaCl-25d, respectively (**Supplementary Table 2**; **Supplementary Figure 5B**). Fifteen DAMs overlapped among the two pairwise comparisons between the two watermelon cultivars under NaCl salt stress for both 2 and 25 days (**Supplementary Figure 5A**).

**Figure 3:**
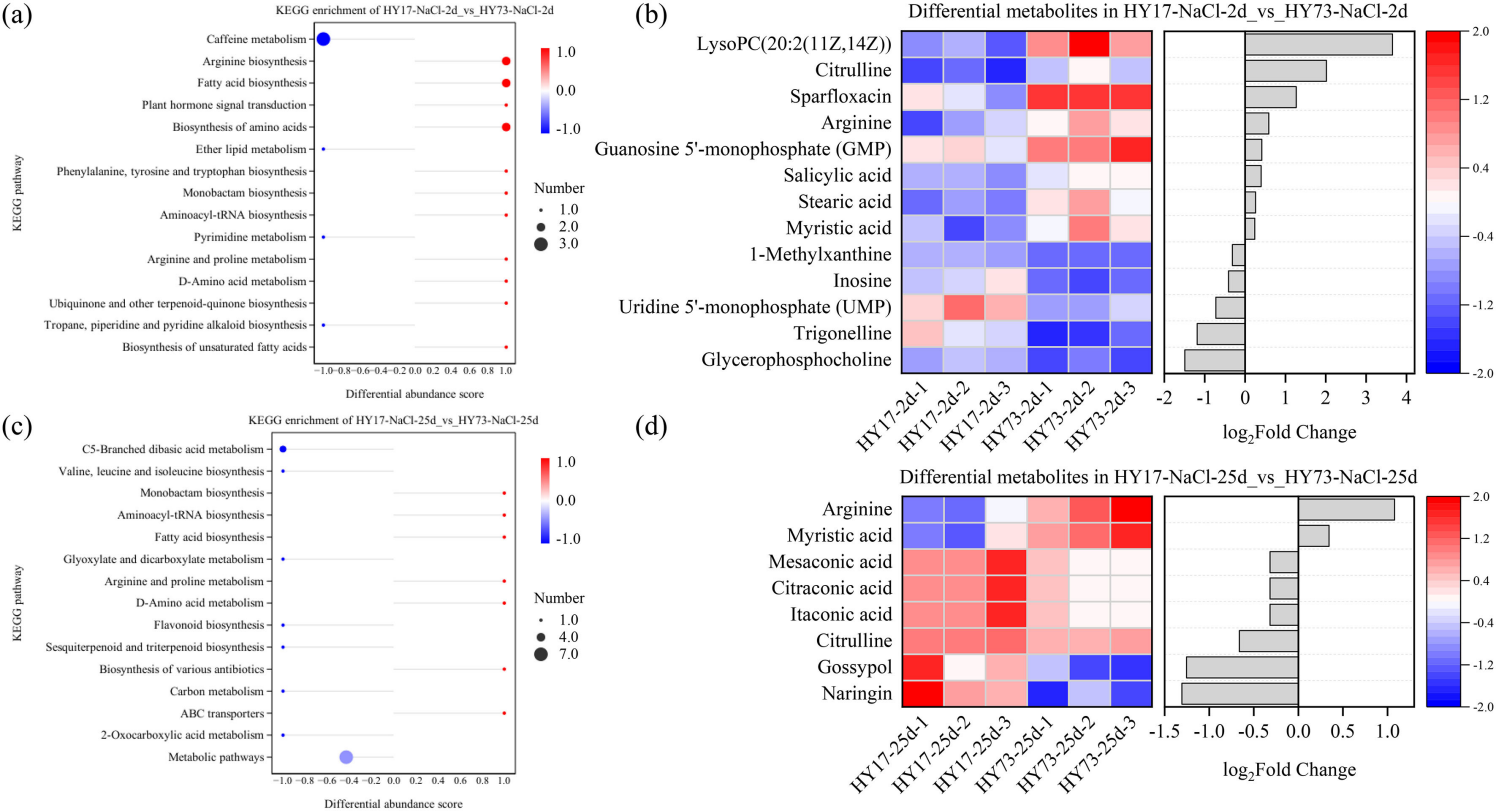
KEGG enrichment of differentially accumulated metabolites (DAMs) in roots between ‘HY17’ and ‘HY73’ watermelon seedlings under salt stress compared by metabolomic analysis. **(a, c)** Differential abundance score of the top 15 KEGG pathways enriched of DAMs between ‘HY17’ and ‘HY73’ watermelon seedlings under salt stress for 2 and 25 days, respectively. **(b, d)** Heatmap of DAMs abundance and differential fold change analysis between ‘HY17’ and ‘HY73’ watermelon seedlings under salt stress for 2 and 25 days, respectively. Differential abundance score = (Number of up-accumulated DAMs – Number of down-accumulated DAMs)/Number of all the DAMs in the pathway.

In the HY17-NaCl-2d_vs_HY73-NaCl-2d pairwise comparison, up-accumulated DAMs in the top 15 KEGG pathways mainly enriched in arginine biosynthesis, fatty acid biosynthesis, plant hormone signal transduction, biosynthesis of amino acids and phenylalanine, and tyrosine and tryptophan biosynthesis, among others, while down-accumulated DAMs in the top 15 KEGG pathways mainly enriched in caffeine metabolism, ether lipid metabolism, pyrimidine metabolism and tropane, and piperidine and pyridine alkaloid biosynthesis (**Figure 3a**; **Supplementary Table 4**). A total of 13 key DAMs were identified from the above KEGG enrichment pathways according to the fold change, namely, 8 up-accumulated DAMs: LysoPC [20:2(11Z,14Z)], citrulline, sparfloxacin, arginine, guanosine 5′-monophosphate (GMP), salicylic acid (SA), stearic acid, and myristic acid, and 5 down-accumulated DAMs: glycerophosphocholine, trigonelline, uridine 5′-monophosphate (UMP), inosine, and 1-methylxanthine (**Figure 3b**).

In the HY17-NaCl-25d_vs_HY73-NaCl-25d pairwise comparison, up-accumulated DAMs in the top 15 KEGG pathways mainly enriched in monobactam biosynthesis, aminoacyl-tRNA biosynthesis, fatty acid biosynthesis, arginine and proline metabolism, and D-amino acid metabolism, among others, while down-accumulated DAMs in the top 15 KEGG pathways mainly enriched in C5-branched dibasic acid metabolism; valine, leucine, and isoleucine biosynthesis; glyoxylate and dicarboxylate metabolism; flavonoid biosynthesis; and sesquiterpenoid and triterpenoid biosynthesis, to name a few (**Figure 3c**; **Supplementary Table 4**). Eight key DAMs were identified from the above KEGG enrichment pathways according to the fold change, including two up-accumulated DAMs—arginine and myristic acid—and six down-accumulated DAMs—naringin, gossypol, citrulline, itaconic acid, citraconic acid, and mesaconic acid (**Figure 3d**).

### 3.3 Integrating transcriptomic and metabolomic analysis of ‘HY17’ and ‘HY73’ under short-term and long-term salt stress

**Figure 4:**
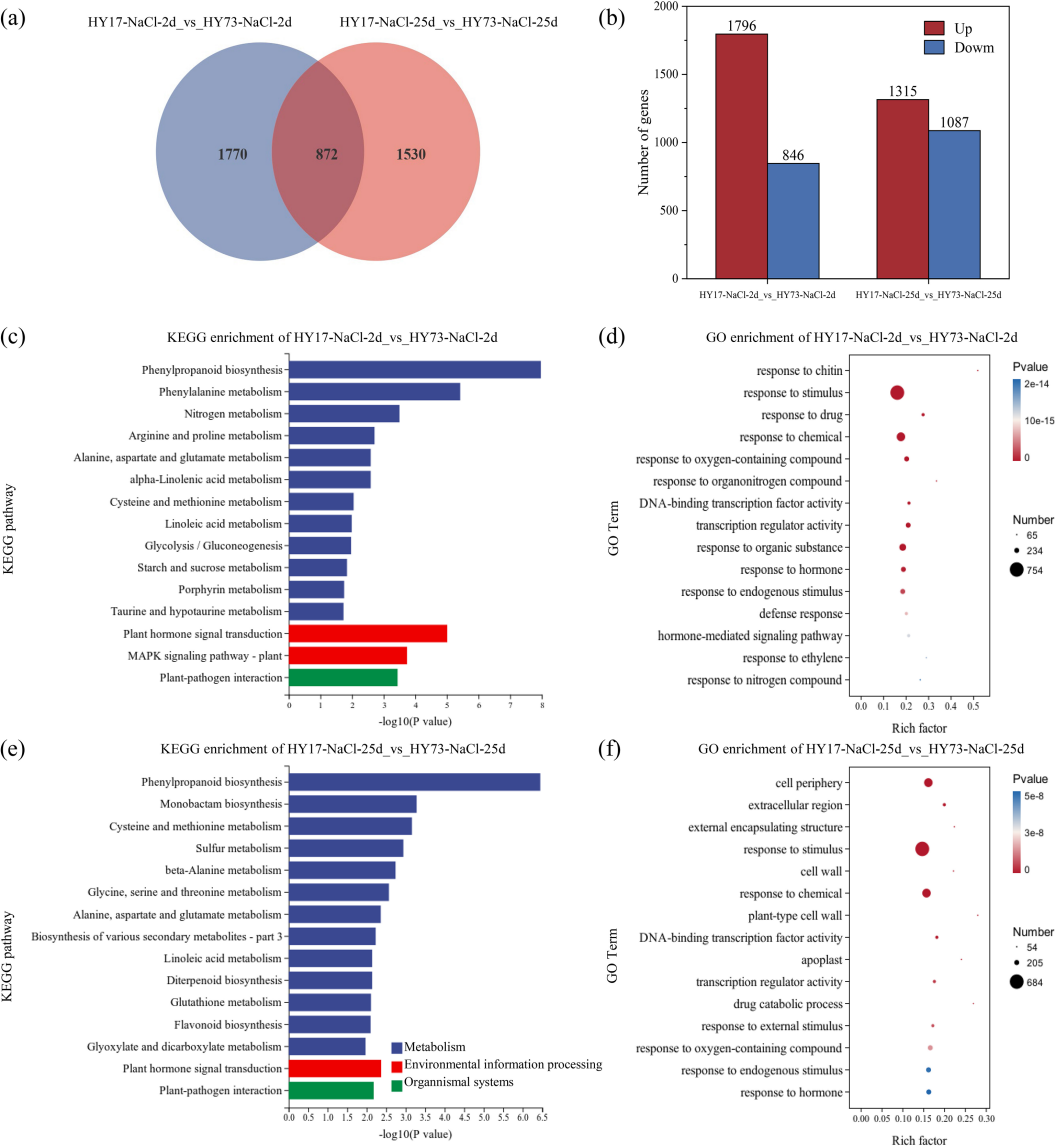
KEGG and GO enrichment of differentially expressed genes (DEGs) in roots between ‘HY17’ and ‘HY73’ watermelon seedlings under salt stress compared by transcriptomic analysis. **(a)** Venn diagram of DEG numbers between ‘HY17’ and ‘HY73’ watermelon seedlings under salt stress for 2 and 25 days. **(b)** Upregulated and downregulated DEG numbers between ‘HY17’ and ‘HY73’ watermelon seedlings under salt stress for 2 and 25 days. **(c, e)** Top 15 KEGG pathways enriched of DEGs between ‘HY17’ and ‘HY73’ watermelon seedlings under salt stress for 2 and 25 days, respectively. **(d, f)** Top 15 GO terms enriched of DEGs between ‘HY17’ and ‘HY73’ watermelon seedlings under salt stress for 2 and 25 days, respectively.

DEGs in roots between ‘HY17’ and ‘HY73’ watermelon seedlings under salt stress for 2 and 25 days, respectively, were identified by comparative transcriptomic analysis. There were 2,642 DEGs (1,796 upregulated and 846 downregulated) between HY17-NaCl-2d_vs_HY73-NaCl-2d pairwise, and 2,402 DEGs (1,315 upregulated and 1,087 downregulated) between HY17-NaCl-25d_vs_HY73-NaCl-25d pairwise (**Supplementary Table 3**; **Figures 4a, b**).

In the HY17-NaCl-2d_vs_HY73-NaCl-2d pairwise comparison, DEGs were enriched in the top 15 KEGG pathways, mainly including phenylpropanoid biosynthesis, phenylalanine metabolism, plant hormone signal transduction, MAPK signaling pathway, nitrogen metabolism, plant–pathogen interaction, arginine and proline metabolism, alanine, aspartate and glutamate metabolism, and alpha-linolenic acid metabolism (**Figure 4c**; **Supplementary Table 5**). GO enrichment of DEGs mainly enriched in terms of response to chitin, response to stimulus, response to drug, response to chemical, response to oxygen-containing compound, and response to organonitrogen compound (**Figure 4d**; **Supplementary Table 6**).

In the HY17-NaCl-25d_vs_HY73-NaCl-25d pairwise comparison, DEGs enriched in the top 15 KEGG pathways mainly including phenylpropanoid biosynthesis; monobactam biosynthesis; cysteine and methionine metabolism; sulfur metabolism; beta-alanine metabolism; glycine, serine, and threonine metabolism; plant hormone signal transduction; and alanine, aspartate, and glutamate metabolism (**Figure 4e**; **Supplementary Table 5**). GO enrichment of DEGs mainly enriched in terms of cell periphery, extracellular region, external encapsulating structure, response to stimulus, cell wall, and response to chemical (**Figure 4f**; **Supplementary Table 6**).

**Figure 5:**
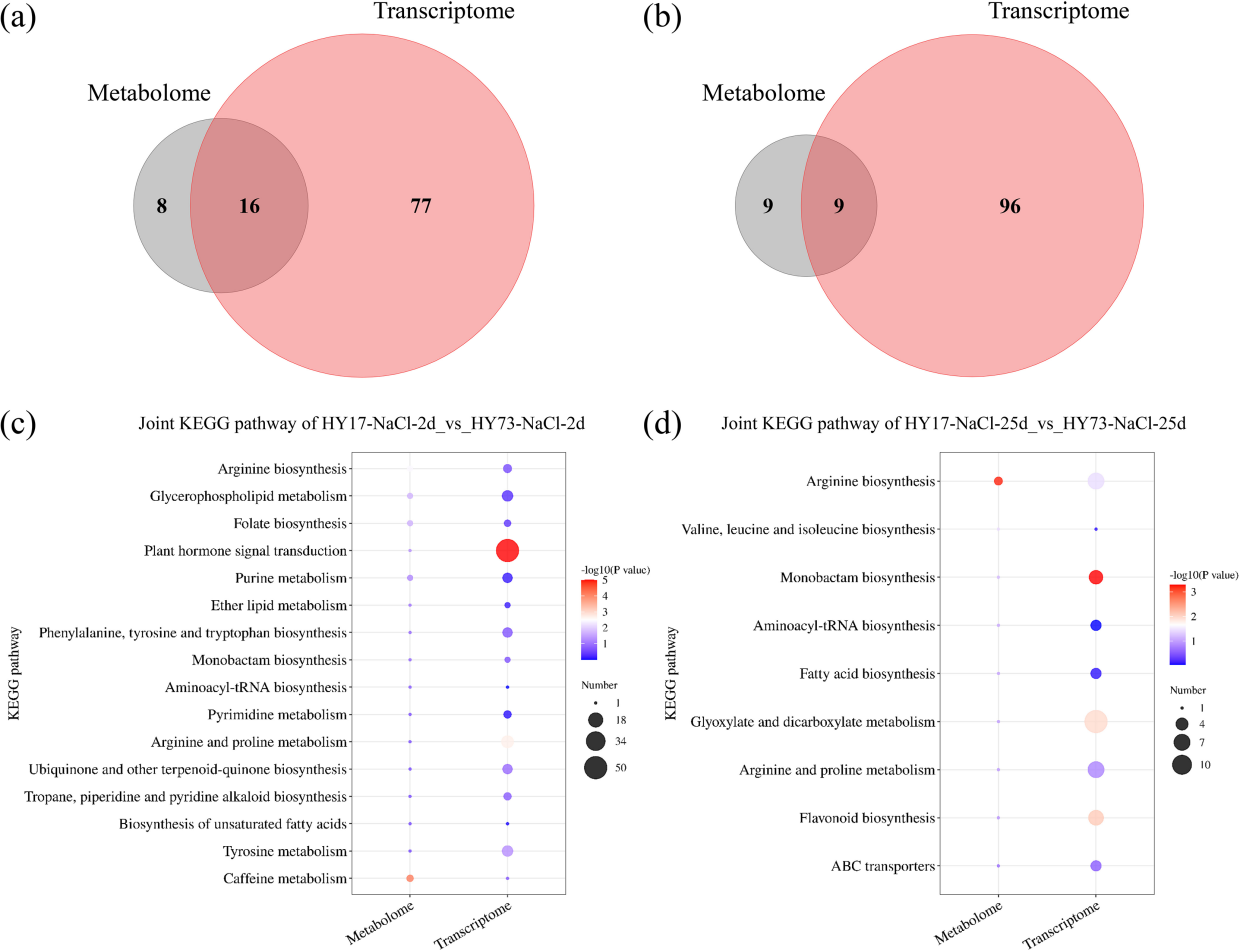
Conjoint analysis of metabolomic and transcriptomic KEGG enrichment between ‘HY17’ and ‘HY73’ watermelon seedlings under salt stress. **(a, b)** Venn diagram of joint KEGG pathway between ‘HY17’ and ‘HY73’ watermelon seedlings under salt stress for 2 and 25 days, respectively. **(c, d)** Joint KEGG pathway of metabolomic and transcriptomic analysis between ‘HY17’ and ‘HY73’ watermelon seedlings under salt stress for 2 and 25 days, respectively.

Furthermore, conjoint analysis of metabolomic and transcriptomic KEGG enrichment between ‘HY17’ and ‘HY73’ watermelon seedlings under salt stress showed that there exist 16 and 9 joint KEGG pathways between the two cultivars under salt stress for 2 and 25 days, respectively (**Figures 5a, b**). The co-enrichment of KEGG analysis indicated that pathways of arginine biosynthesis and plant hormone signal transduction are crucial for response to 2 days of short-term salt stress, and the arginine biosynthesis pathway also plays a role in tolerance to 25 days of long-term salt stress (**Figures 5c, d**).

### 3.4 Salicylic acid derived from the phenylalanine metabolism pathway plays an important role in the short-term response to salt stress

**Figure 6:**
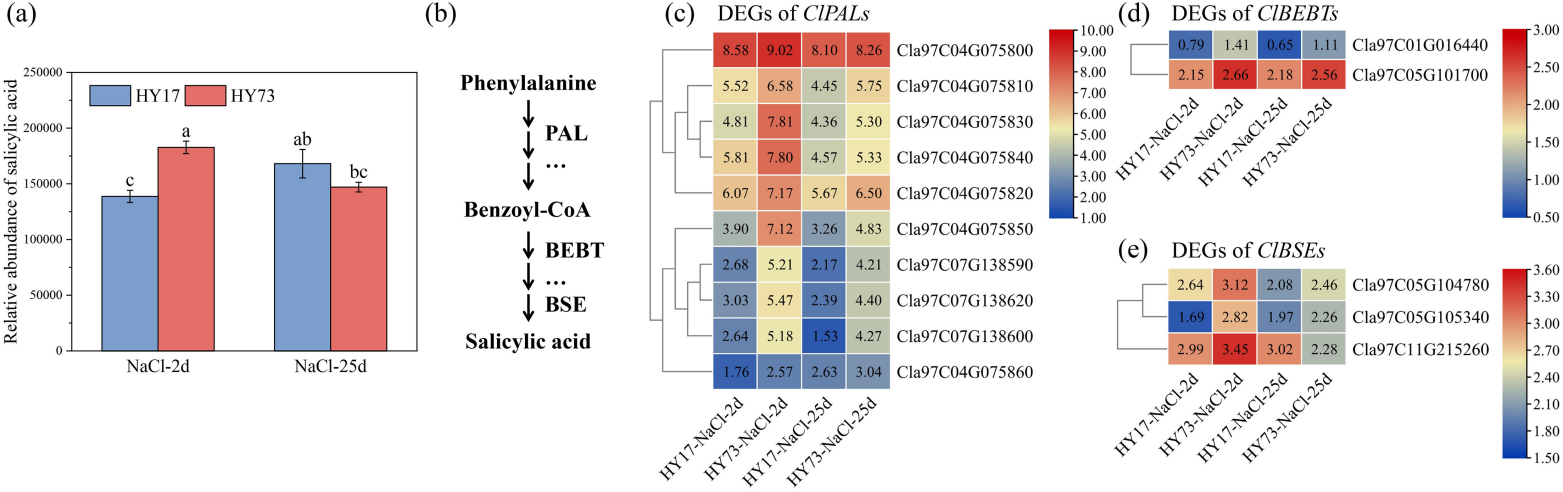
Salicylic acid biosynthesis pathway differentially expressed genes analysis between “HY17” and “HY73” watermelon seedlings under salt stress. **(a)** Relative abundance of salicylic acid in roots under salt stress for 2 and 25 days. Values are the means ± SD of three biological replicates (*n* = 3), and different letters on each column indicate significant differences between means among different groups at the *p* < 0.05 level compared by one-way ANOVA with Duncan test. **(b)** Pathway of salicylic acid biosynthesis. PAL: phenylalanine ammonia-lyase; BEBT: benzyl alcohol O-benzoyltransferase; BSE: benzylsalicylate esterase. **(c)** Heatmap of ClPALs differential expression. **(d)** Heatmap of ClBEBTs differential expression. **(e)** Heatmap of ClBSEs differential expression. The gene expression level of TPM is log_2_-transformed.

SA is the only differentially accumulated plant hormone identified from the comparative metabolome between ‘HY17’ and ‘HY73’ seedlings under salt stress (**Figure 3b**). SA content in the salt-tolerant cultivar ‘HY73’ was significantly higher than that in the salt-sensitive cultivar ‘HY17’ after 2 days of salt stress (fold change = 1.317). It was demonstrated that SA plays an important role in the short-term response to salinity in watermelon (**Figure 6a**). SA biosynthesis was derived from phenylalanine metabolism (**Figure 6b**). It can also be validated from the KEGG pathway enrichment of DEGs between HY17-NaCl-2d_vs_HY73-NaCl-2d pairwise, with the top two KEGG pathways being phenylpropanoid biosynthesis and phenylalanine metabolism in this study (**Figure 4c**). In the SA biosynthesis pathway, 10 DEGs of phenylalanine ammonia-lyase (ClPALs), 2 DEGs of benzyl alcohol O-benzoyltransferase (ClBEBTs), and 3 DEGs of benzylsalicylate esterase (ClBSEs) were screened out, which contribute to SA accumulation response to short-term salt stress in ‘HY73’ watermelon seedling (**Figures 6c–e**).

### 3.5 Arginine and myristic acid accumulation plays a role in the long-term tolerance to salt stress

**Figure 7:**
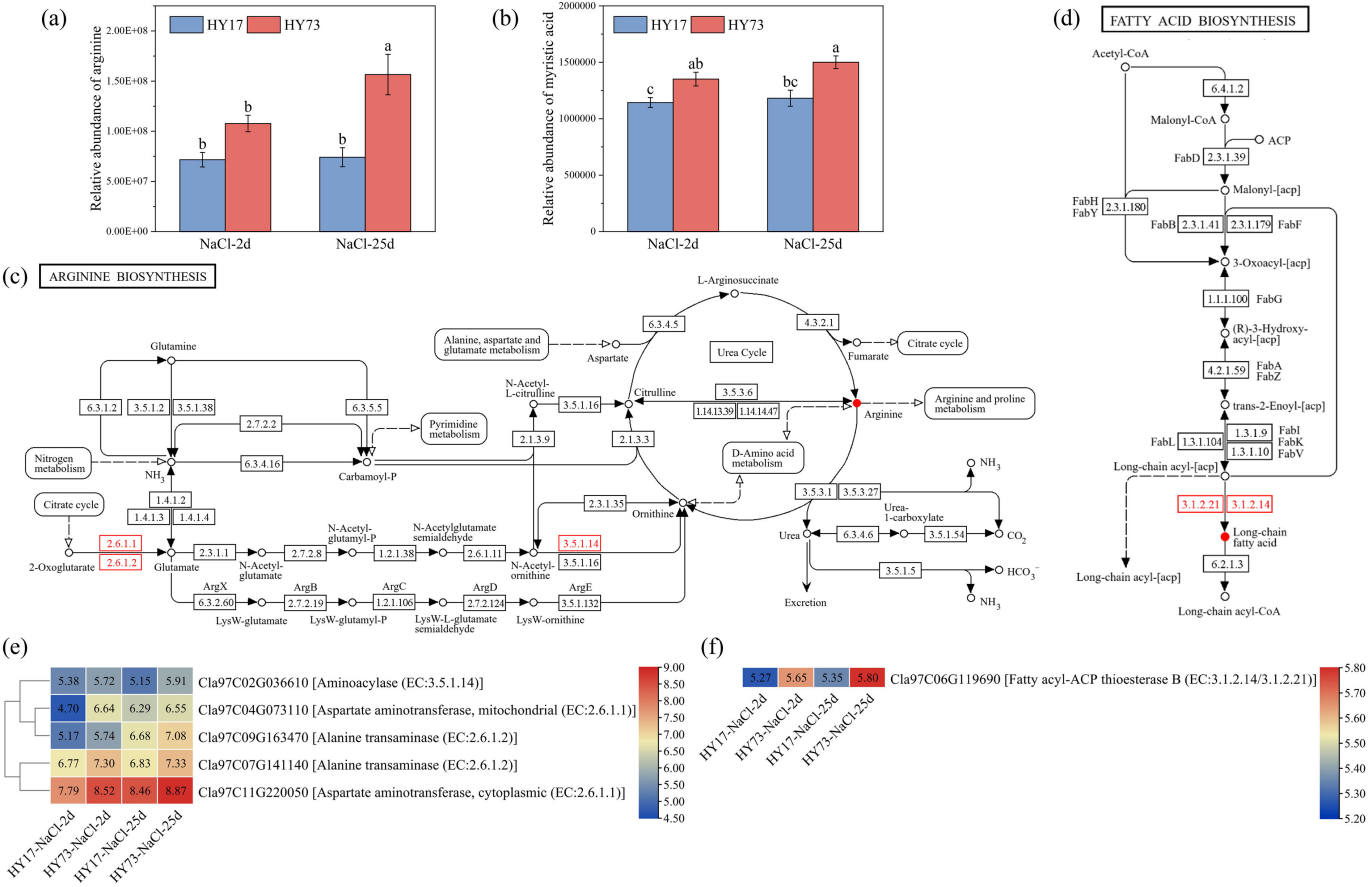
Arginine biosynthesis and fatty acid biosynthesis pathway differentially expressed gene analysis between ‘HY17’ and ‘HY73’ watermelon seedlings under salt stress. **(a)** Relative abundance of arginine in roots under salt stress for 2 and 25 days. **(b)** Myristic acid content in roots under salt stress for 2 and 25 days. Values are the means ± SD of three biological replicates (*n* = 3), and different letters on each column indicate significant differences between means among different groups at the *p* < 0.05 level compared by one-way ANOVA with Duncan test. **(c)** Pathway of arginine biosynthesis. **(d)** Pathway of myristic acid biosynthesis. **(e)** Heatmap of differentially expressed genes in arginine biosynthesis pathway. **(f)** Heatmap of differentially expressed genes in myristic acid biosynthesis pathway. The gene expression level of TPM is log_2_-transformed.

According to the results of comparative metabolomic analysis, arginine and myristic acid consistently accumulated at higher levels in the salt-tolerant cultivar ‘HY73’ than in the salt-sensitive cultivar ‘HY17’ under salt stress for 2 and 25 days, with a fold change of 1.503 and 2.113 for arginine, and with a fold change of 1.182 and 1.270 for myristic acid, respectively (**Figures 7a, b**). This suggests that the two metabolites play a role in the long-term tolerance to salinity in watermelon. In the arginine biosynthesis pathway, five DEGs of aminoacylase (ClACYs) (Cla97C02G036610), aspartate aminotransferase (ClASTs) (Cla97C11G220050 and Cla97C04G073110), and alanine transaminase (ClALTs) (Cla97C09G163470 and Cla97C07G141140), respectively, were screened out (**Figures 7c, d**). In the fatty acid biosynthesis pathway, one DEG of fatty acyl-ACP thioesterase B (ClFATBs) (Cla97C06G119690) was screened out (**Figures 7d, f**). These genes are the key DEGs of arginine and myristic accumulation for salt stress tolerance of ‘HY73’ watermelon seedlings.

### 3.6 Na^+^ and K^+^ accumulation and the salt overly sensitive pathway of ‘HY17’ and ‘HY73’ under salt stress

Na^+^ and K^+^ share the transport channel of high-affinity K^+^ transporter (HKT) and low-affinity K^+^ transporter (KT); therefore, the Na^+^/K^+^ homeostasis is always disrupted due to the excessive Na^+^ uptake to compete with K^+^ transportation under salinity. In addition, the SOS pathway is essential for regulating the intracellular Na^+^ level in plant under salinity. This pathway involves the cascade collaboration of three proteins: SOS3 (Calcineurin B-like, CBL), a calcium-binding protein; SOS2 (CBL-interacting protein kinase, CIPK), a serine/threonine protein kinase; and SOS1 (Na^+^/H^+^ exchanger, NHX), a plasma membrane-localized Na^+^/H^+^ antiporter.

Accumulation of Na^+^ and K^+^ in shoots and roots of ‘HY17’ and ‘HY73’ watermelon seedlings showed that ‘HY73’ had significantly lower Na^+^ levels in roots and shoots than ‘HY17’ under salt stress for 25 days. The K^+^/Na^+^ ratios in roots of ‘HY17’ and ‘HY73’ were 0.50 and 0.61, respectively. The K^+^/Na^+^ ratios in shoots of ‘HY17’ and ‘HY73’ were 1.52 and 2.23, respectively. Moreover, the [Na^+^]_shoot_/[Na^+^]_root_ ratios of ‘HY17’ and ‘HY73’ were 0.71 and 0.48, respectively. These results indicated that the salt-tolerant cultivar ‘HY73’ accumulated less Na^+^ than the salt-sensitive cultivar ‘HY17’ under salinity (**Table 2**).

**Table 2:** Accumulation of Na^+^ and K^+^ in shoots and roots of ‘HY17’ and ‘HY73’ watermelon seedlings under salt stress for 25 days.

Treatments	Shoots			Roots			[Na^+^]_shoot_/[Na^+^]_root_
	Na^+^ (mg·g^−1^ DW)	K^+^ (mg·g^−1^ DW)	K^+^/Na^+^	Na^+^ (mg·g^−1^ DW)	K^+^ (mg·g^−1^ DW)	K^+^/Na^+^	
HY17-Control	1.40 ± 0.18d	48.25 ± 0.51a	34.35 ± 0.67a	6.92 ± 0.07c	32.13 ± 0.14a	4.64 ± 0.06b	0.20 ± 0.01d
HY17-NaCl	23.95 ± 0.21a	36.46 ± 0.21c	1.52 ± 0.02c	33.63 ± 0.12a	16.71 ± 0.89d	0.50 ± 0.01c	0.71 ± 0.01a
HY73-Control	1.83 ± 0.07c	45.47 ± 0.48b	24.80 ± 0.70b	5.84 ± 0.13d	31.22 ± 0.14b	5.35 ± 0.14a	0.31 ± 0.01c
HY73-NaCl	15.76 ± 0.16b	35.08 ± 0.27d	2.23 ± 0.03c	33.10 ± 0.33b	20.12 ± 0.34c	0.61 ± 0.01c	0.48 ± 0.01b

Values are the means ± SD of three biological replicates (*n* = 3), and different letters represent significant differences at the level of *p* < 0.05.

**Figure 8:**
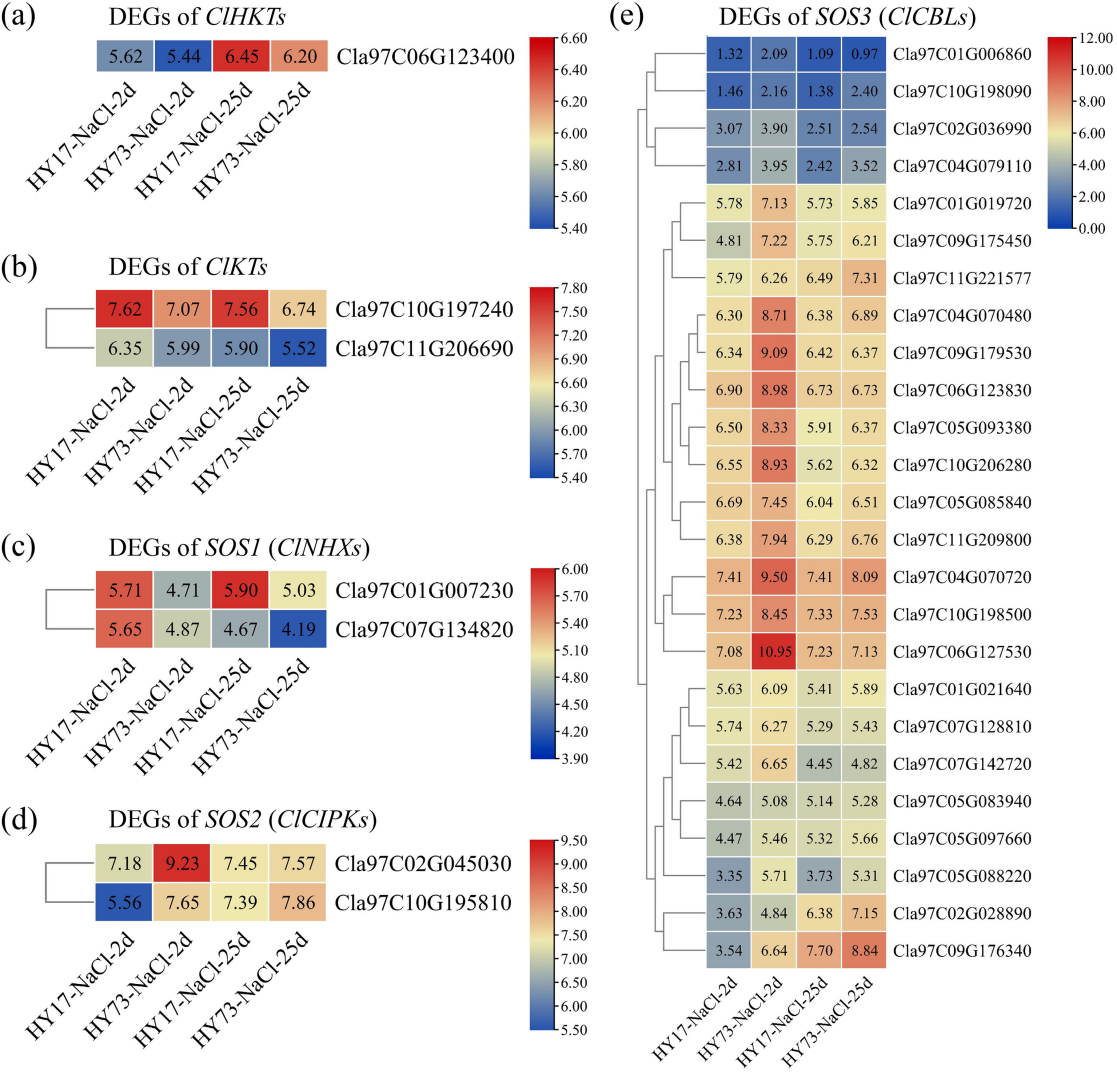
Na^+^ and K^+^ transport and salt overly sensitive pathway differentially expressed gene analysis between ‘HY17’ and ‘HY73’ watermelon seedlings under salt stress. **(a)** Heatmap of ClHKTs differential expression. **(b)** Heatmap of ClKTs differential expression. **(c)** Heatmap of SOS1 (ClNHXs) differential expression. **(d)** Heatmap of SOS2 (ClCIPKs) differential expression. **(e)** Heatmap of SOS3 (ClCBLs) differential expression. The gene expression level of TPM is log_2_-transformed. HKT, high-affinity K^+^ transporter; KT, low-affinity K^+^ transporter; NHX, Na^+^/H^+^ exchanger; CIPK, CBL-interacting protein kinase; CBL, calcineurin B-like.

On the one hand, one DEG of ClHKTs (Cla97C06G123400) and two DEGs of ClKTs (Cla97C10G197240 and Cla97C11G206690) associated with cellular K^+^ and Na^+^ uptake were found; they are all significantly lower expressed in ‘HY73’ than in ‘HY17’, leading to less Na^+^ intake (**Figures 8a, b**). On the other hand, 2 DEGs of SOS2 (ClCIPKs) and 25 DEGs of SOS3 (ClCBLs) associated with intracellular Na^+^ expulsion were identified; they are all significantly higher expressed in ‘HY73’ than in ‘HY17’, leading to more Na^+^ expulsion by activating the SOS pathway. The highly expressed SOS2 member Cla97C02G045030 and SOS3 member Cla97C06G127530 in ‘HY73’ are key genes for salt signal perception and transduction in the watermelon SOS pathway (**Figures 8c–e**).

## 4 Discussion

### 4.1 Salicylic acid induces plant stress resistance and responds to salinity

SA is a well-known phenol-based plant defense hormone (Ding and Ding, 2020; Ma et al., 2025a). SA has a multifaceted role in regulating systemic resistance processes, including photosynthesis, nitrogen metabolism, antioxidant defense system, and osmotic adjustment (Khan et al., 2015). The imperative role of SA in ameliorating salt stress has been tested by applying exogenous SA and its analogs on various plants (Singh et al., 2022). It has been reviewed that the exogenous application of SA can significantly alleviate a salt-induced decrease in photosynthesis by increasing antioxidant activity to reduce oxidative damage to protect the photosynthesis system and maintain an optimum osmotic homeostasis by promoting ionic acquisition and the synthesis of osmoprotectants in many plant species (Jayakannan et al., 2015; Zhao et al., 2020).

**Figure 9:**
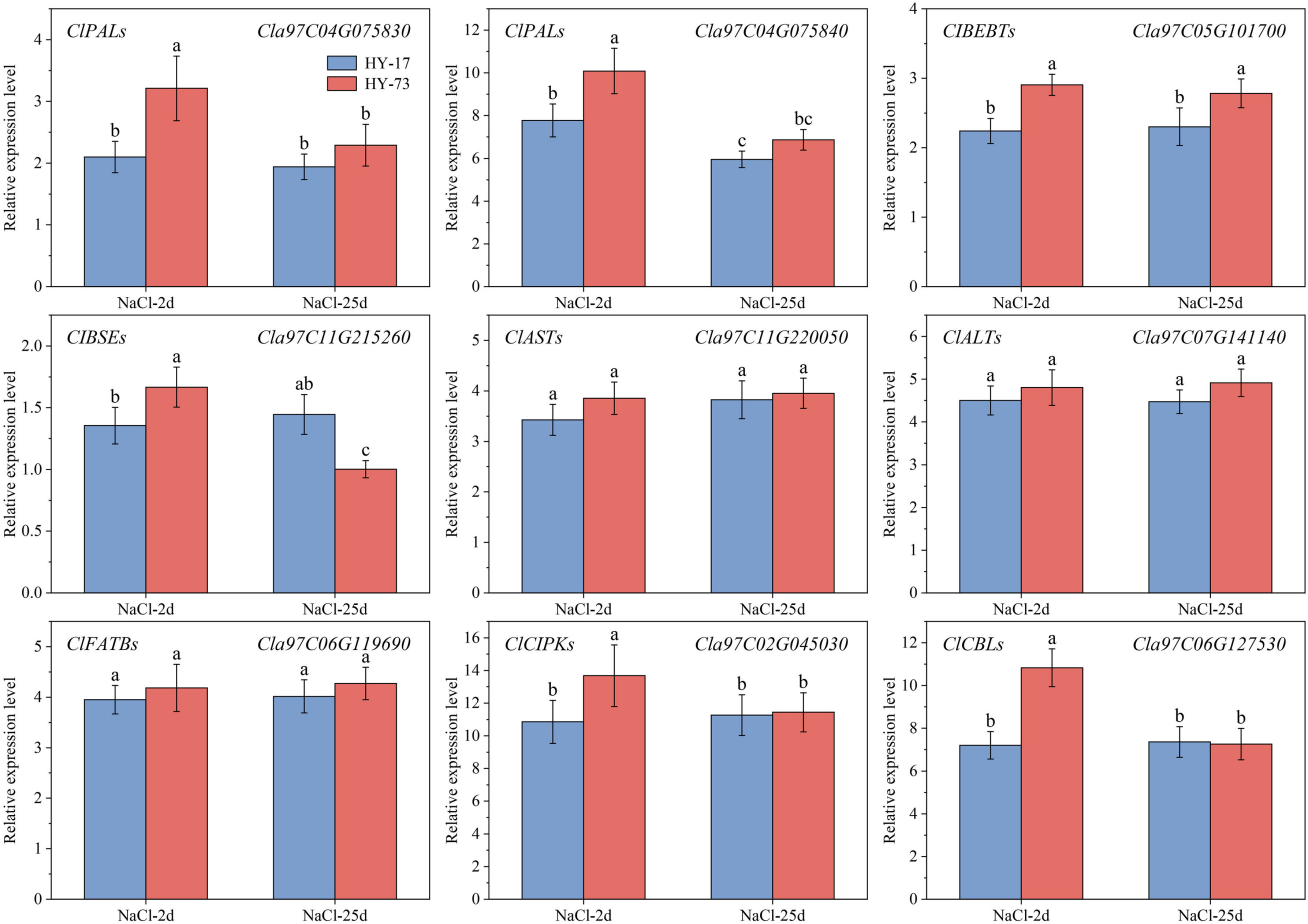
RT-qPCR expression validation of selected key DEGs associated with salinity resistance in watermelon. DEGs in salicylic acid biosynthesis pathway (ClPALs, Cla97C04G075830 and Cla97C04G075840; CIBEBTs, Cla97C05G101700; CIBSEs, Cla97C11G215260); DEGs in arginine biosynthesis pathway (ClASTs, Cla97C11G220050; ClALTs, Cla97C07G141140); DEGs in fatty acid biosynthesis pathway (ClFATBs, Cla97C06G119690); DEGs in salt overly sensitive (SOS) pathway (ClCIPKs, Cla97C02G045030; ClCBLs, Cla97C06G127530). Values are the means ± SD of three replicates, and different letters on each column indicate significant differences between means among different groups at the *p* < 0.05 level compared by one-way ANOVA with Duncan test.

Furthermore, SA could also ameliorate salinity stress by counteracting salt stress-induced membrane depolarization and by decreasing K^+^ efflux. In *Arabidopsis*, SA pretreatment enhanced the activity of H^+^-ATPase, decreased NaCl-induced membrane depolarization, and minimized NaCl-induced K^+^ leakage from the cell within the first hour under salt stress. In long-term NaCl treatments, SA increased shoot K^+^ and decreased shoot Na^+^ accumulation (Jayakannan et al., 2013). Furthermore, SA might also crosstalk with other hormones, such as gibberellins (GA), abscisic acid (ABA), jasmonic acid (JA), and ethylene (ETH), which are correlated with the activation of osmotic adjustment and the maintenance of ionic homeostasis for salt tolerance (Jayakannan et al., 2015). RT−qPCR expression validation of selected key DEGs in the SA biosynthesis pathway in this study is shown in **Figure 9**.

### 4.2 Arginine might be a novel indicator for salt tolerance genotype selection of watermelon

Amino acids are a type of vital osmoprotectants that are accumulated in plants during stress conditions. These amino acids decrease the osmotic potential of cells, allowing water absorption, stabilize protein structures and membranes, and also act as nitrogen storing agents and ROS scavengers under salinity (Singh et al., 2022). However, the amino acid components accumulated in cucurbits in response to salinity seem special (Malambane et al., 2023). Wild watermelon has been found to primarily accumulate citrulline, and then glutamate and arginine, in place of proline and glycine betaine (Kawasaki et al., 2000). Furthermore, watermelon is the model organism for citrulline accumulation under stress especially for drought tolerance (Song et al., 2020). Plants often encounter salt stress along with desiccation during drought stress; as the water content in the soil decreases, the salt content increases. However, the response of watermelons to salinity was different from their response to drought, showing that the citrulline accumulated much later after the start of drought stress, while changes in the amino acid content and composition were very rapid under salt stress with less citrulline accumulation than under drought in leaves (Yokota et al., 2002). In addition, from the arginine biosynthesis pathway, we can learn that arginine and citrulline can convert into each other with several enzymes involved (**Figure 7c**). In this study, a higher citrulline accumulation was also observed in the salt-tolerant cultivar ‘HY73’ than in the salt-sensitive cultivar ‘HY17’ under 2 days of salt stress, but became lower than that of ‘HY17’ under 25 days of salt stress (**Figures 3a, c**). Therefore, arginine and citrulline might be considered as novel biochemical indicators for salt tolerance genotype selection of watermelon.

In addition, exogenous arginine plays diverse roles in mitigating salt stress damage. In wheat, arginine treatments significantly mitigated the negative impacts of salinity on growth and productivity via enhancing photosynthetic pigments, accumulated osmoprotectants, total soluble sugars (TSS), free amino acids, and proline; enhancing indole acetic acid (IAA) content and phenolic biosynthesis; and reducing oxidative damage and lipid peroxidation through triggered antioxidant enzymes (Li et al., 2024; Ragaey et al., 2022). Furthermore, exogenous arginine significantly reduced the concentrations of Na^+^ and the Na^+^/K^+^ ratio, while significantly increasing K^+^ levels, thereby preserving ionic osmotic balance in plants under salinity (Li et al., 2024; Nahar et al., 2025).

In a study using the watermelon inbred line “TN07011” cultured by nutrient solution treated with 150 mM NaCl, leaves that were fully expanded from the two to three growth points were collected for metabolomic and transcriptomic analysis. DAMs and DEGs enrichments showed that watermelon seedlings responded to salt stress mainly by regulating amino acid- and lipid metabolism-related pathways. Seven key DAMs [L-glutamate, 4-aminobutanoate (GABA), proline, L-asparagine, L-aspartate, L-alanine, and citrulline] were observed to be up-accumulated, and 21 DEGs related to amino acid metabolism were found, including the ClASTs genes (Cla97C04G073110 and Cla97C11G220050), which were also found in our study (**Figure 7e**). However, no significant differences were found in arginine content in the study, which may be due to the tissue difference in sampling between leaves and roots compared to our study (Liu et al., 2023). RT−qPCR expression validation of selected key DEGs in the arginine biosynthesis pathway in this study is shown in **Figure 9**.

### 4.3 Myristic acid plays a roles in alleviating membrane lipid peroxidation and SOS3 N-myristoylation

Under salt stress, the accumulation of ROS leads to membrane lipid peroxidation damage, causing loss of fluidity and selective permeability of the cellular and organelle membrane system. As the main product of membrane lipid peroxidation reaction, MDA was usually used to evaluate the degree of membrane lipid overoxidation (Roxas et al., 2000). The MDA content in tissues of leaves and roots of ‘HY17’ and ‘HY73’ watermelon seedlings significantly increased under salt conditions, but ‘HY73’ experienced a slightly higher degree of membrane lipid peroxidation than ‘HY17’, accompanied by a higher myristic acid accumulation in roots, especially under long-term salt stress for 25 days (**Figures 2D, E**, **8B**). Fatty acids could serve as lipid membrane protectants to prevent oxidative damage. Qureshi et al. (2013) revealed a salt-induced shift toward long-chain and monosaturated fatty acids through fatty-acid profiling in *Artemisia annua* L. Under a salinity of 160 mM NaCl, myristic acid, palmitoleic acid, linoleic acid, and erucic acid content increased by 141%, 186%, 34% and 908%, respectively. While oleic acid, linolenic acid, arachidonic acid, and lignoceric acid content decreased by 50%, 17%, 44%, and 78%, respectively. Thus, modification in fatty acid composition might be a membrane lipid adaptation to oxidative stress under long-term salinity.

In addition, myristic acid plays an important role in protein N-myristoylation, which refers to the covalent modification of myristic acid by an amide bond to the N-terminal glycine residue of a nascent polypeptide. In most cases, this modification can promote proteins to mediate membrane association or protein–protein interaction (Johnson et al., 1994). Therefore, it was hypothesized that myristic acid is required for the N-myristoylation of SOS3 (CIPKs) function in regulating the sodium detoxification signaling pathways for plant salt tolerance (Ishitani et al., 2000). RT−qPCR expression validation of selected key DEGs in the fatty acid biosynthesis pathway in this study is shown in **Figure 9**.

### 4.4 Key SOS2 and SOS3 members in the salt overly sensitive pathway of watermelon

The SOS pathway is important for regulating intracellular Na^+^ homeostasis in plants in response to salinity. Salt stress inducing a rapid elevation in cytosolic Ca^2+^ level is sensed by SOS3, which can bind to and activate the SOS2 kinase, and then the SOS3–SOS2 kinase complex interacts with and triggers SOS1 to expel Na^+^ out of the cell or storge into the vacuole, thus preventing Na^+^ accumulation in the cytoplasm under salinity (Ali et al., 2023).

However, the watermelon gene families of the SOS pathway have not been identified. In this study, a total of 30 SOS1 (ClNHXs) members, 20 SOS2 (ClCIPKs) members, and 110 SOS3 (ClCBLs) members were identified genome-wide in watermelon (**Supplementary Table 1**). Comparative transcriptomic analysis of DEGs in the SOS pathway between the roots of ‘HY17’ and ‘HY73’ watermelon seedlings under salinity indicated that ‘HY73’ has more sensitive salt-signal perception and transduction ability in activating the SOS pathway to expel Na^+^ than ‘HY17’ under short-term (2 days) salt stress; the SOS2 member Cla97C02G045030 and the SOS3 member Cla97C06G127530 are key members responsible for this mechanism (**Figures 8d, e**). RT−qPCR expression validation of the two genes is shown in **Figure 9**. Additionally, a lower expression level of Na^+^/K^+^ transport proteins ClHKTs (Cla97C06G123400) and ClKTs (Cla97C10G197240 and Cla97C11G206690) limited the Na^+^ influx in ‘HY73’ to prevent excessive salt stress from the beginning (**Figures 8a, b**). Meanwhile, a lower expression level of ClHKTs gene Cla97C06G123400 was also observed in the comparison between the seedlings of the salt-tolerant watermelon cultivar “Zhongshihong” and the salt-sensitive watermelon variety “PI186489” after 7 days of 150 mM NaCl treatment in roots (Zhu et al., 2022).

## 5 Conclusions

**Figure 10:**
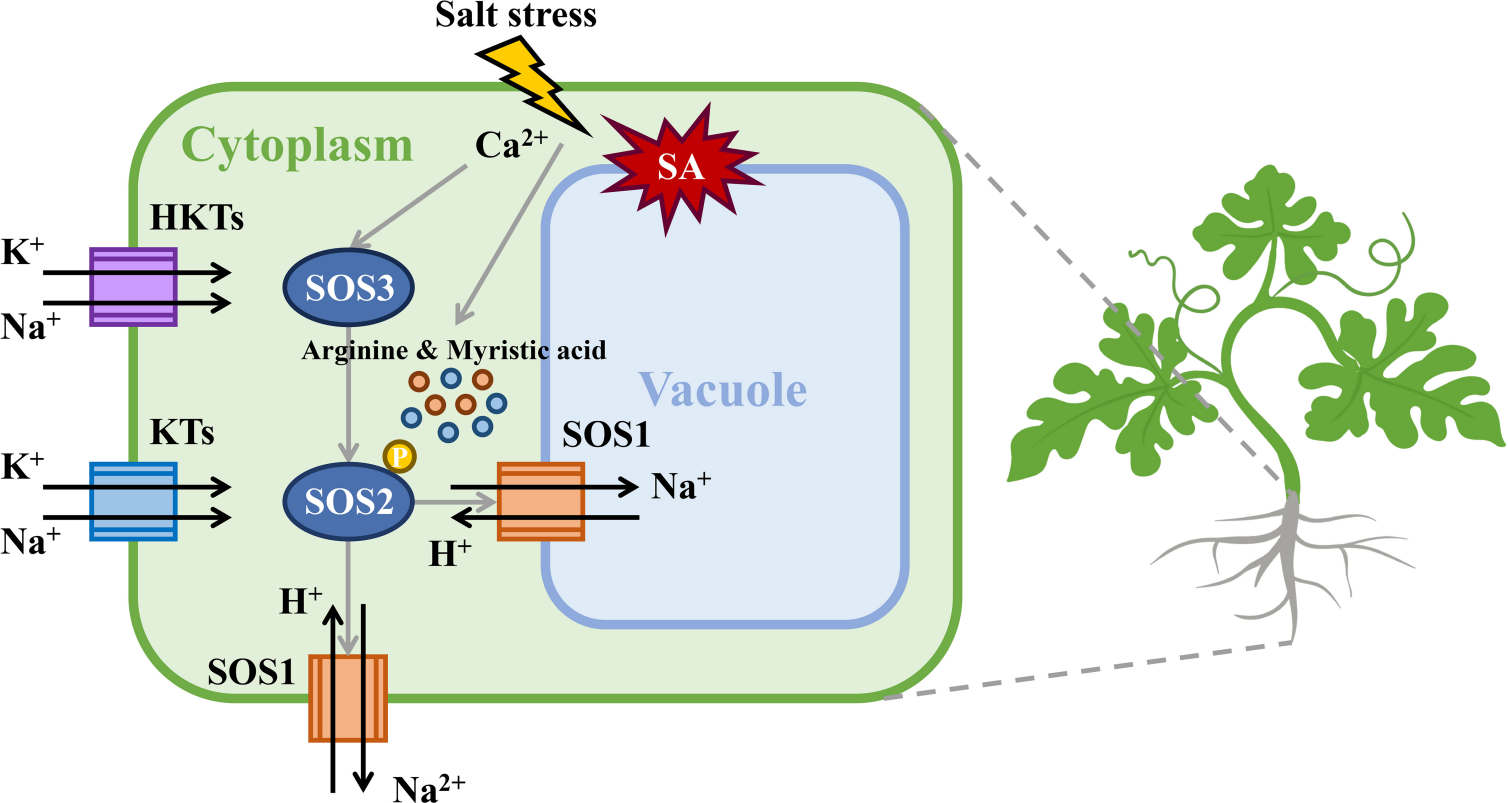
Mechanisms of response and tolerance to salt stress in watermelon. SA, salicylic acid; HKT, high-affinity K^+^ transporter; KT, low-affinity K^+^ transporter; SOS1 (NHXs), Na^+^/H^+^ exchangers; SOS2 (CIPKs), CBL-interacting protein kinases; SOS3 (CBLs), calcineurin B-likes.

Watermelon cultivars salt-sensitive ‘HY17’ and salt-tolerant ‘HY73’ were used as materials in this study. Watermelon seedlings were treated with 150 mM NaCl for 2 and 25 days, and roots were sampled for comparative metabolomic and transcriptomic analysis. Results revealed that more SA synthesized from the phenylalanine metabolism pathway plays a role in rapid response to short-term salt stress in ‘HY73’ than in ‘HY17’. On the other hand, higher arginine and myristic acid accumulation in ‘HY73’ than in ‘HY17’ is crucial for tolerance to long-term salt stress related to ROS scavenging and alleviating membrane lipid peroxidation. DEGs of ClASTs (Cla97C04G073110 and Cla97C11G220050) and ClALTs (Cla97C09G163470 and Cla97C07G141140) for arginine biosynthesis, and fatty acyl-ACP thioesterase B gene (Cla97C06G119690) for myristic acid biosynthesis were screened out to improve salt tolerance. Additionally, DEGs of ClHKTs (Cla97C06G123400) and ClKTs (Cla97C10G197240) were key to limiting Na^+^ intake for salinity resistance. Key members of ClCIPKs (Cla97C02G045030) and ClCBLs (Cla97C06G127530) in the SOS pathway were identified as responsible for intracellular K^+^/Na^+^ homeostasis under salt stress. These findings provide important metabolic foundations and candidate gene targets for salt-tolerant watermelon breeding (**Figure 10**).

## Data Availability

The data presented in the study are deposited in the NCBI SRA (https://www.ncbi.nlm.nih.gov/sra) repository, accession number PRJNA1429590.
