# Supplementary material for: Comparative metabolomic and transcriptomic analysis revealing the role of salicylic acid for short-term response with arginine and myristic acid for long-term tolerance under salt stress in watermelon (Citrullus lanatus L.)
Source: Front Plant Sci. 2026 Jun 26;17:1823719. doi: 10.3389/fpls.2026.1823719 (PMC13352413; doi:10.3389/fpls.2026.1823719)
Supplement: Supplementary file 7 [file DataSheet1.docx]

**Comparative metabolomic and transcriptomic analysis revealing the role of salicylic acid for short-term response with arginine and myristic acid for long-term tolerance under salt stress in watermelon (*****Citrullus lanatus* L.)**


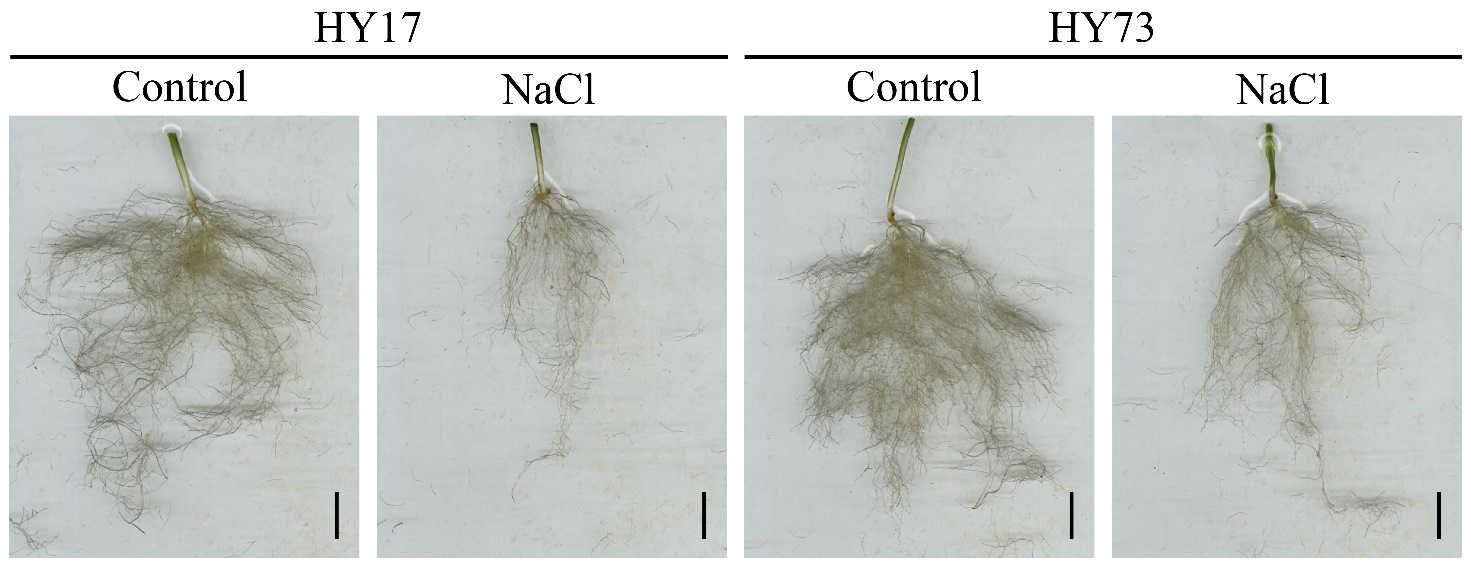


**FIGURE S1** Roots performance of ‘HY17’ and ‘HY73’ watermelon seedlings under salt stress for 25 days. Bar=2 cm.


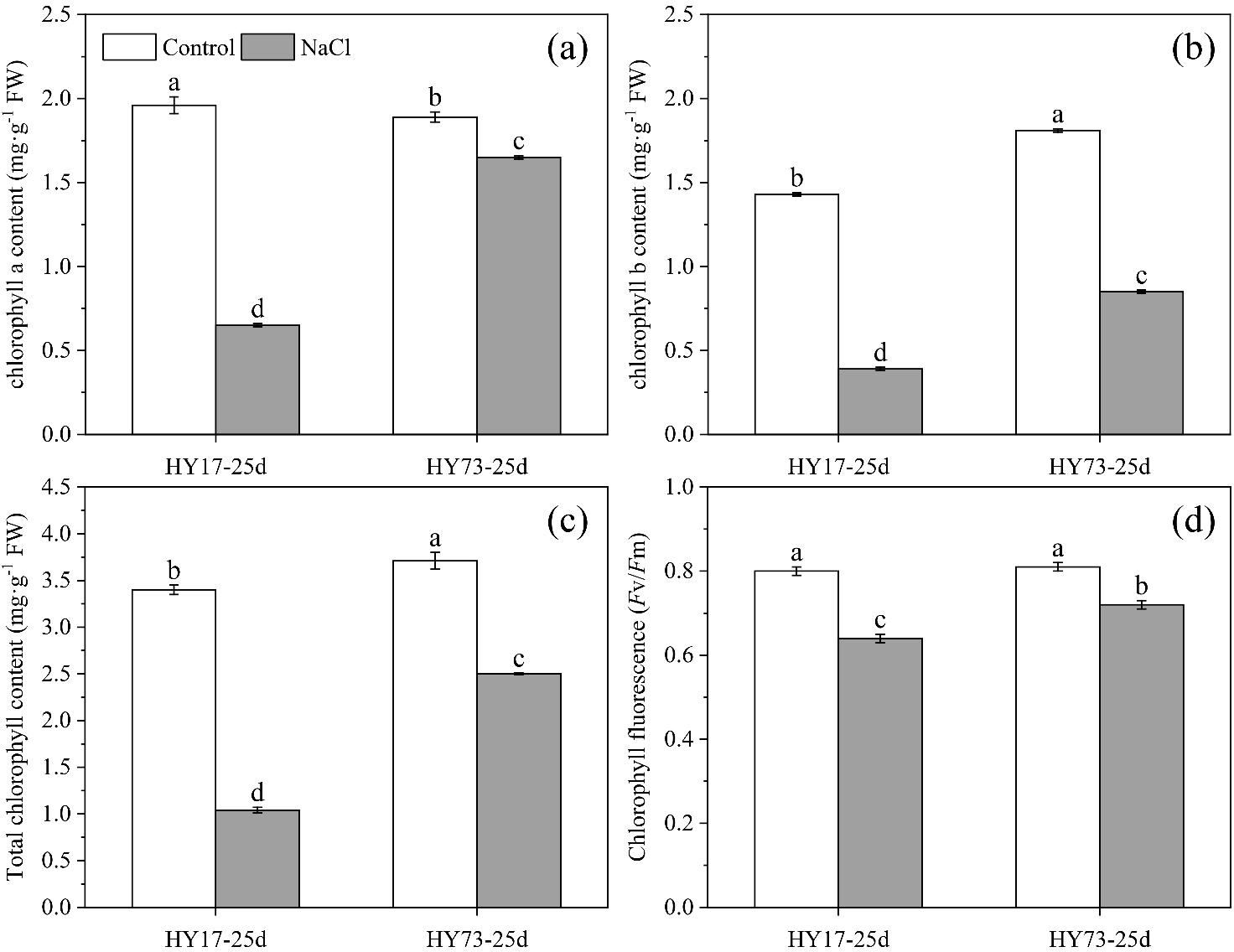


**FIGURE S2** Leaf chlorophyll content and chlorophyll fluorescence of ‘HY17’ and ‘HY73’ watermelon seedlings under salt stress for 25 days. (a) Chlorophyll a content in leaves. (b) Chlorophyll b content in leaves. (c) Total chlorophyll content in leaves. (d) Chlorophyll fluorescence of maximal photochemical efficiency of PS-II (*F*v/*F*m) of leaves. Values are the means ± SD of three biological replicates (n = 3), and different letters on each column indicate significant differences between means among different groups at *p* < 0.05 level compared by One-way ANOVA with Duncan test.


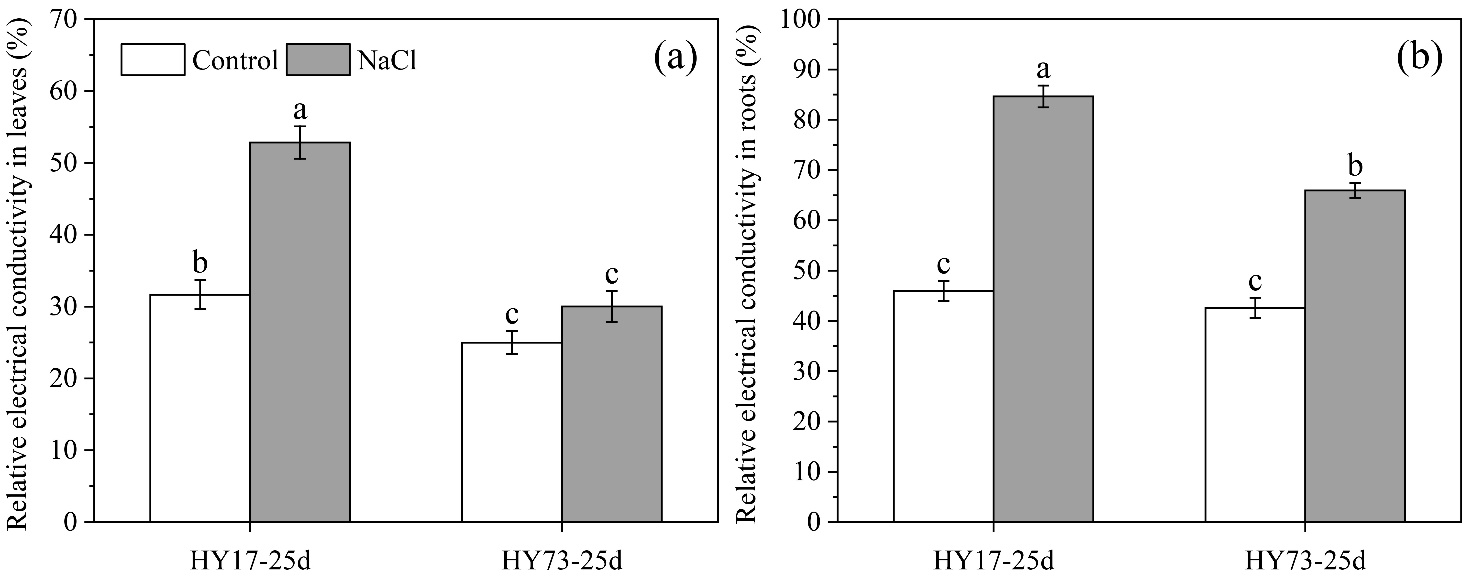


**FIGURE S3** Relative electrical conductivity in leaves and roots of ‘HY17’ and ‘HY73’ watermelon seedlings under salt stress for 25 days. (a) Relative electrical conductivity in leaves. (b) Relative electrical conductivity in roots. Values are the means ± SD of three biological replicates (n = 3), and different letters on each column indicate significant differences between means among different groups at *p* < 0.05 level compared by One-way ANOVA with Duncan test.


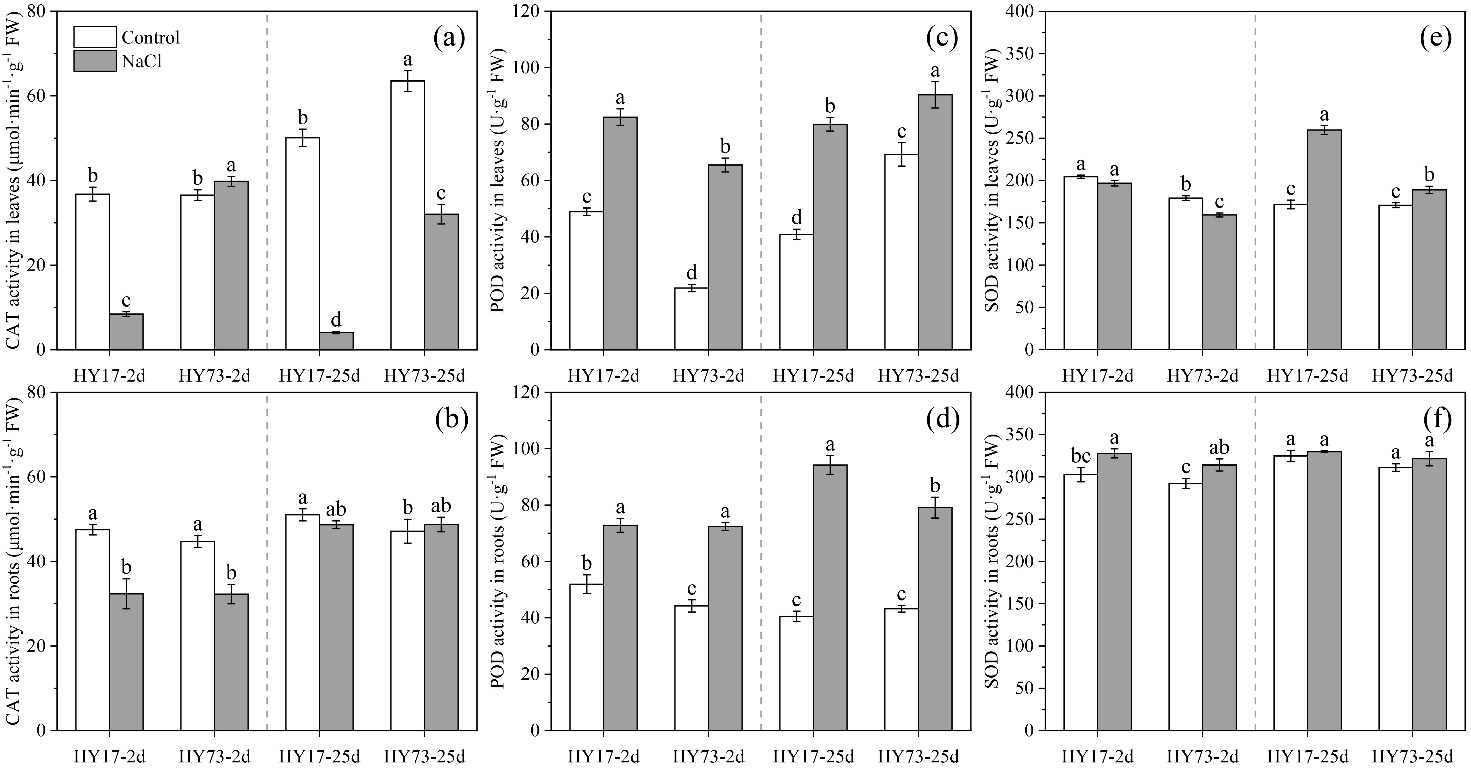


**FIGURE S4** Antioxidant enzymes activity in shoots and roots of HY17 and HY73 watermelon seedlings under salt stress. (a, b) Catalase (CAT) activities in leaves and roots under salt stress for 2 days and 25 days. (c, d) Peroxidase (POD) activities in leaves and roots under salt stress for 2 days and 25 days. (e, f) Superoxide dismutase (SOD) activities in leaves and roots under salt stress for 2 days and 25 days. Values are the means ± SD of three biological replicates (n = 3), and different letters on each column indicate significant differences between means among different groups at *p* < 0.05 level compared by One-way ANOVA with Duncan test.


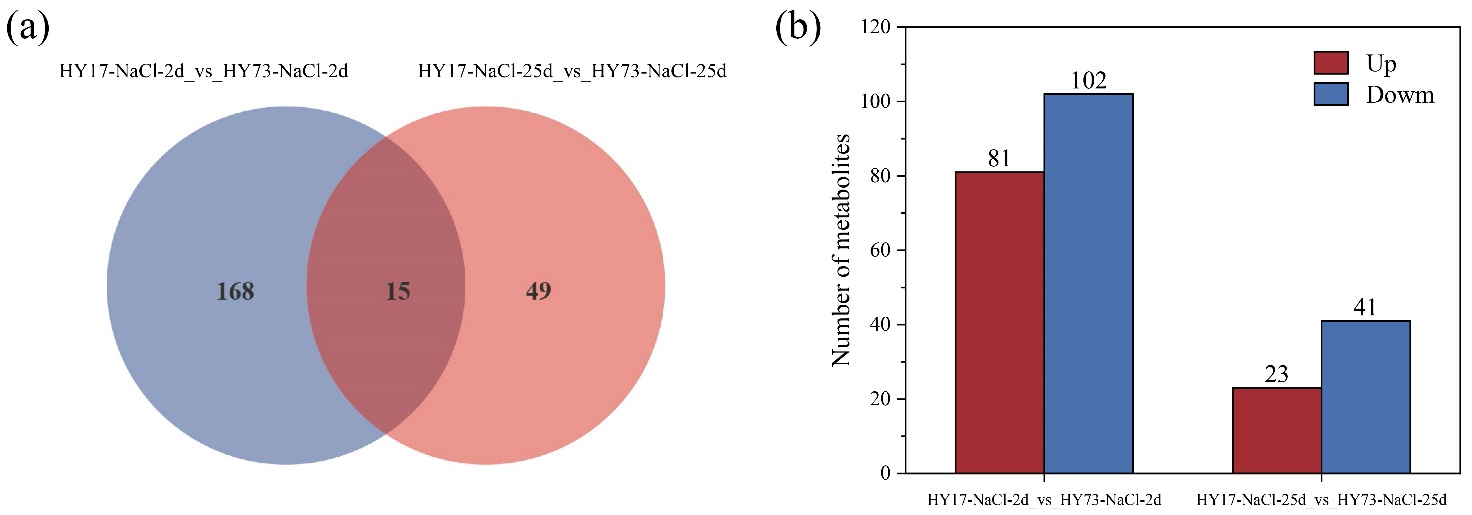


**FIGURE S5** Differentially accumulated metabolites (DAMs) in roots compared by metabolomic analysis between ‘HY17’ and ‘HY73’ watermelon seedlings under salt stress. (a) Venn diagram of DAMs number between ‘HY17’ and ‘HY73’ watermelon seedlings under salt stress for 2 days and 25 days. (b) Up-regulated and down-regulated DAMs number between ‘HY17’ and ‘HY73’ watermelon seedlings under salt stress for 2 days and 25 days.


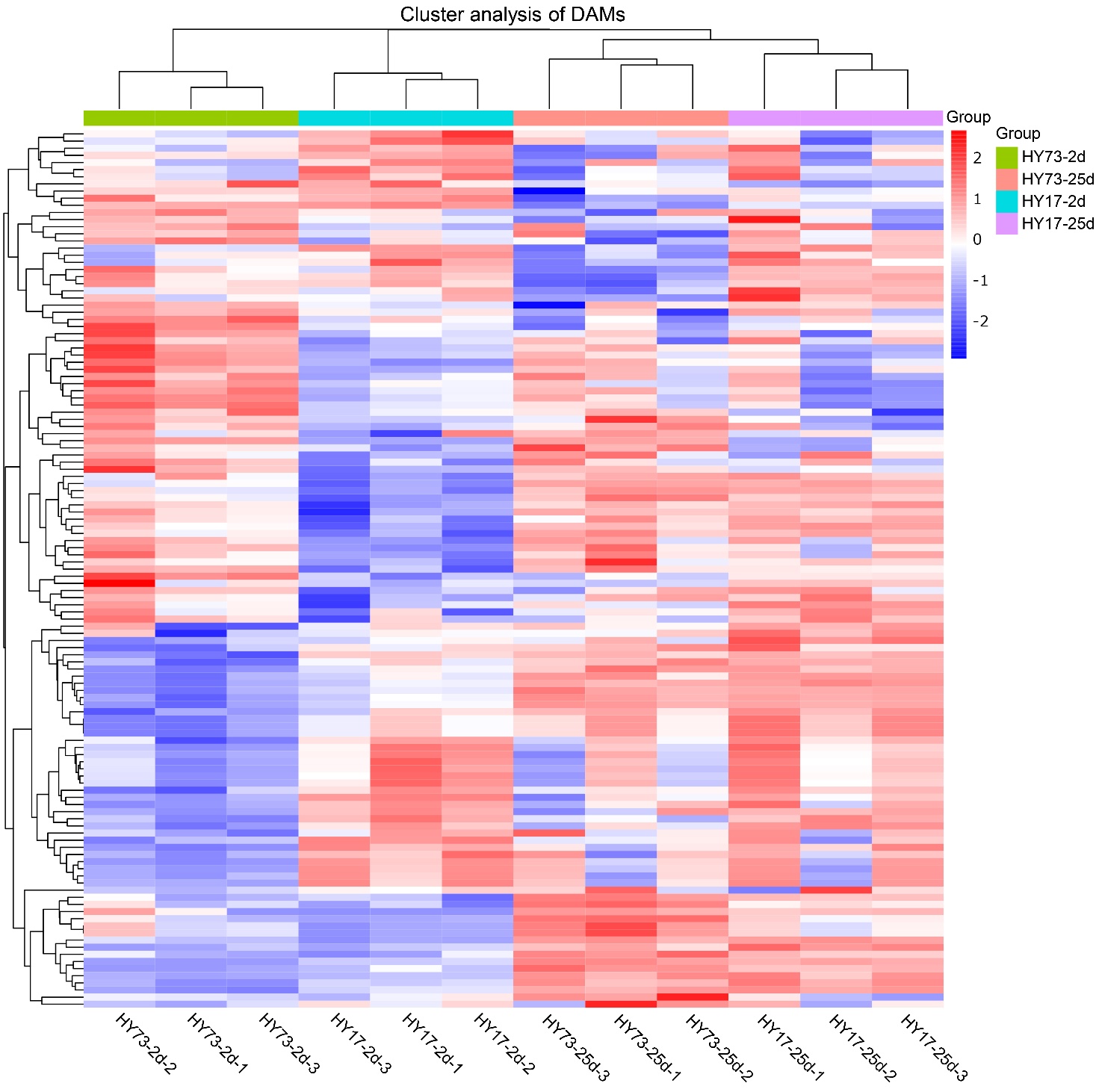


**FIGURE S6** Cluster analysis of differentially accumulated metabolites (DAMs) in roots between ‘HY17’ and ‘HY73’ watermelon seedlings under salt stress compared by metabolomic analysis.


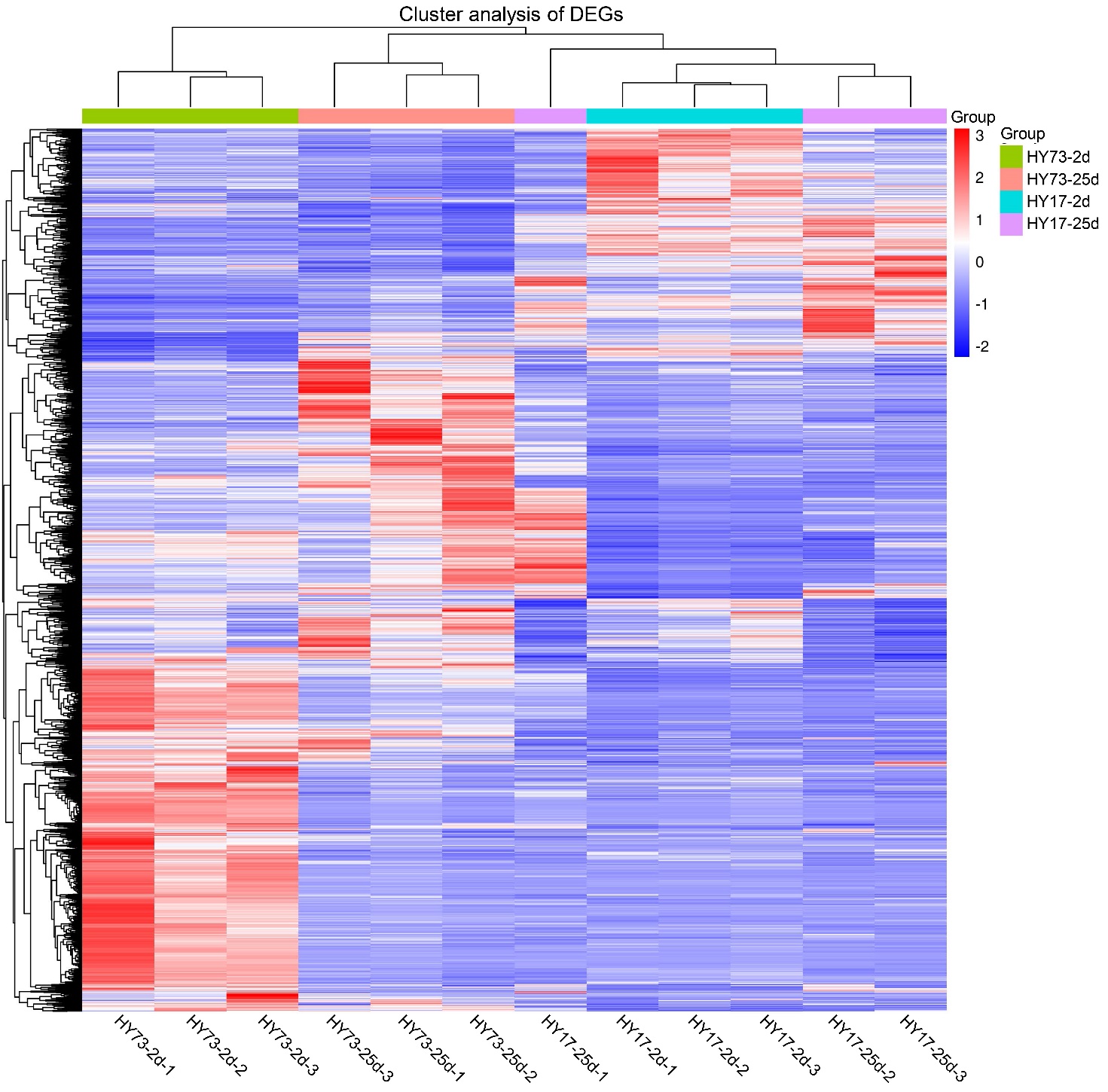


**FIGURE S7** Cluster analysis of differentially expressed genes (DEGs) in roots between ‘HY17’ and ‘HY73’ watermelon seedlings under salt stress compared by transcriptomic analysis.

**Table S1** *ClHKTs*, *ClKTs*, *ClNHXs*, *ClCIPKs*, *ClCBLs* family members identified in watermelon.

**Table S2** Differentially accumulated metabolites (DAMs) in roots between ‘HY17’ and ‘HY73’ watermelon seedlings under salt stress.

**Table S3** Differentially expressed genes (DEGs) in roots between ‘HY17’ and ‘HY73’ watermelon seedlings under salt stress.

**Table S4** KEGG enrichment analysis of differentially accumulated metabolites (DAMs) in roots between ‘HY17’ and ‘HY73’ watermelon seedlings under salt stress.

**Table S5** KEGG enrichment analysis of differentially expressed genes (DEGs) in roots between ‘HY17’ and ‘HY73’ watermelon seedlings under salt stress.

**Table S6** GO enrichment analysis of differentially expressed genes (DEGs) in roots between ‘HY17’ and ‘HY73’ watermelon seedlings under salt stress.
